# Analysis of essential genes in *Clostridioides difficile* by CRISPRi and Tn-seq

**DOI:** 10.1128/jb.00220-25

**Published:** 2025-09-08

**Authors:** Maia E. Alberts, Micaila P. Kurtz, Ute Müh, Jonathon P. Bernardi, Kevin W. Bollinger, Horia A. Dobrila, Leonard Duncan, Hannah M. Laster, Andres J. Orea, Anthony G. Pannullo, Juan G. Rivera-Rosado, Facundo V. Torres, Craig D. Ellermeier, David S. Weiss

**Affiliations:** 1 Department of Microbiology and Immunology, Carver College of Medicine, University of Iowa 12243 , Iowa City, Iowa, USA; 2 Graduate Program in Genetics, University of Iowa, Iowa City, Iowa, USA; The Ohio State University, Columbus, Ohio, USA

**Keywords:** CRISPRi, Tn-seq

## Abstract

Essential genes are interesting in their own right and as potential antibiotic targets. To date, only one report has identified essential genes on a genome-wide scale in *Clostridioides difficile*, a problematic pathogen for which treatment options are limited. That foundational study used large-scale transposon mutagenesis to identify 404 protein-encoding genes as likely to be essential for vegetative growth of the epidemic strain R20291. Here, we revisit the essential genes of strain R20291 using a combination of CRISPR interference (CRISPRi) and transposon insertion site sequencing (Tn-seq). First, we targeted 181 of the 404 putatively essential genes with CRISPRi. We confirmed essentiality for >90% of the targeted genes and observed morphological defects for >80% of them. Second, we conducted a new Tn-seq analysis, which identified 346 genes as essential, of which 283 are in common with the previous report and might be considered a provisional essential gene set that minimizes false positives. We compare the list of essential genes to those of other bacteria, especially *Bacillus subtilis*, highlighting some noteworthy differences. Finally, we used fusions to red fluorescent protein (RFP) to identify 18 putative new cell division proteins, 3 of which are conserved in Bacillota but of largely unknown function. Collectively, our findings provide new tools and insights that advance our understanding of *C. difficile*.

**IMPORTANCE:**

*Clostridioides difficile* is an opportunistic pathogen for which better antibiotics are sorely needed. Most antibiotics target pathways that are essential for viability. Here, we use saturation transposon mutagenesis and gene silencing with CRISPR interference to identify and characterize genes required for growth on laboratory media. Comparison to the model organism *Bacillus subtilis* revealed many similarities and a few striking differences that warrant further study and may include opportunities for developing antibiotics that kill *C. difficile* without decimating the healthy microbiota needed to keep *C. difficile* in check.

## INTRODUCTION

*Clostridioides difficile* infections (CDI) kill close to 13,000 people a year in the United States (1). Treating CDI is challenging because the antibiotics effective against *C. difficile* also impact the normal intestinal microbiota needed to keep *C. difficile* in check (2–4). There is a need for improved antibiotics that inhibit *C. difficile* more selectively. Most clinically useful antibiotics target proteins or pathways that are essential for viability, so a deeper understanding of the essential genes in *C. difficile* might provide foundational knowledge to guide antibiotic development. Essential genes are also interesting in their own right, as they provide insights into the most fundamental aspects of bacterial physiology.

 Transposon insertion site sequencing (Tn-seq) identifies essential genes on a genome-wide scale based on the absence of insertions following saturation transposon mutagenesis (5, 6). However, several caveats must be kept in mind when interpreting the output of a Tn-seq experiment. For instance, insertion mutants that are viable but grow slowly will be lost from the mutant pool during outgrowth, so some apparently essential genes can be deleted. This caveat underscores the fact that binary categorization of genes as essential or non-essential is useful but an oversimplification. Tn-seq might also erroneously classify non-essential genes as essential due to polarity onto *bona fide* essential genes or because the random nature of Tn insertions means genes might be missed for stochastic reasons. Finally, Tn-seq does not provide insight into the actual function of essential genes because the phenotypic defects of the corresponding insertion mutants are not observed. Despite these caveats and limitations, Tn-seq is a powerful tool for prioritizing genes to investigate by more laborious methods.

CRISPR interference (CRISPRi) is a complementary approach for genome-wide interrogation of essential genes in bacteria (7–12). CRISPRi uses a single-guide RNA (sgRNA) to direct a catalytically inactive Cas9 protein (dCas9) to a gene of interest, thereby repressing transcription (13). As the organism continues to grow and divide, it becomes depleted of the targeted protein, potentially revealing phenotypic changes that precede cell death. Thus, CRISPRi provides functional information that Tn-seq cannot. However, CRISPRi shares with Tn-seq the problem of polarity, which has to be taken into consideration when interpreting phenotypes.

In 2015, Dembek et al. used Tn-seq to identify 404 protein-encoding genes as essential for vegetative growth in *C. difficile* strain R20291 on BHI media (14). As expected, most of these genes encode proteins involved in core biological processes and cell surface biogenesis, but some are of unknown function or not expected to be essential. Here, we revisit the essential genes of strain R20291 using a combination of CRISPRi and Tn-seq. First, we targeted 181 of the 404 putatively essential genes with CRISPRi to vet essentiality and identify terminal phenotypes. We confirmed essentiality for >90% of the targeted genes and observed morphological defects for >80% of them. Second, we conducted a new and more thorough Tn-seq analysis to identify genes essential for vegetative growth on TY media. We classified 346 protein-coding genes as essential, of which 283 (~80%) were also essential in the previous study. Finally, we conducted a microscopy-based screen to identify potential cell division proteins. We discuss our findings in light of what is known about essential genes and cell division in other bacteria, particularly *Bacillus subtilis*.

## RESULTS AND DISCUSSION

### A library for CRISPRi knockdown of 181 putative essential genes

Our *C. difficile* CRISPRi plasmid has been described (15). It expresses a codon-optimized *dCas9* from a xylose-inducible promoter (P_xyl_) and a sgRNA from a constitutively active glutamate dehydrogenase promoter (P_gdh_). Constructing a knockdown library involved several steps: selecting the genes to be targeted, designing the sgRNAs, cloning those sgRNAs into the CRISPRi plasmid, and moving the finished plasmids from *E. coli* into *C. difficile* by conjugation. Because conjugation efficiencies are low, plasmids have to be moved from *E. coli* into *C. difficile* one by one. This step imposes a bottleneck that makes it impractical to target all 404 essential genes identified previously. We therefore trimmed the gene list by excluding all transposon and phage-related genes (because these are not part of the core genome), most genes for tRNA synthetases and ribosomal proteins (to limit redundancy), and most genes for small proteins, defined here as fewer than 80 amino acids (240 nucleotides). Short genes are small targets for Tn insertion, so a disproportionate fraction is likely to be false positives. At this point, we were left with 252 genes. Because CRISPRi is polar (7, 13, 16, 17), there is little to be gained by targeting multiple genes in an operon, so, in most cases, we targeted only one gene per transcription unit as annotated in BioCyc (v28.5, release Dec 2024) (18).

In the end, we selected a total of 181 putatively essential genes for CRISPRi knockdown (Table S1). We constructed a library of individual sgRNA clones, using two sgRNAs per gene for a total of 362 CRISPRi plasmids (Table S2). Although our experiments were restricted to strain R20291, 91% of sgRNAs are perfect matches to the corresponding genes in strain 630 as well (Table S2). As negative controls, we constructed 20 CRISPRi plasmids with scrambled sgRNAs that do not target anywhere in the R20291 genome (Table S2). Plasmids were confirmed by sequencing across the P_gdh_::sgRNA element in *E. coli* and after conjugation into *C. difficile*. Of the genes targeted for knockdown, 86 have an essential ortholog in *Bacillus subtilis*, 62 have a non-essential ortholog in *B. subtilis*, and 33 have no *B. subtilis* ortholog, including four hypothetical genes. However, the number of genes of unknown function is larger than four because many of the non-hypotheticals have homology to domains with such broadly or ill-defined functions that it is not obvious what these genes do or why they would be essential (e.g., “glycosyltransferase,” “two-component response regulator,” or “DUF1846”). Considering that 110 of the targeted genes are predicted to be in operons with other apparently essential genes, our study encompasses 281 of the 404 genes identified as essential by Tn mutagenesis, close to 70% of the total (14).

### Essentiality determined by CRISPRi knockdown largely agrees with Tn-seq data

**Fig 1:**
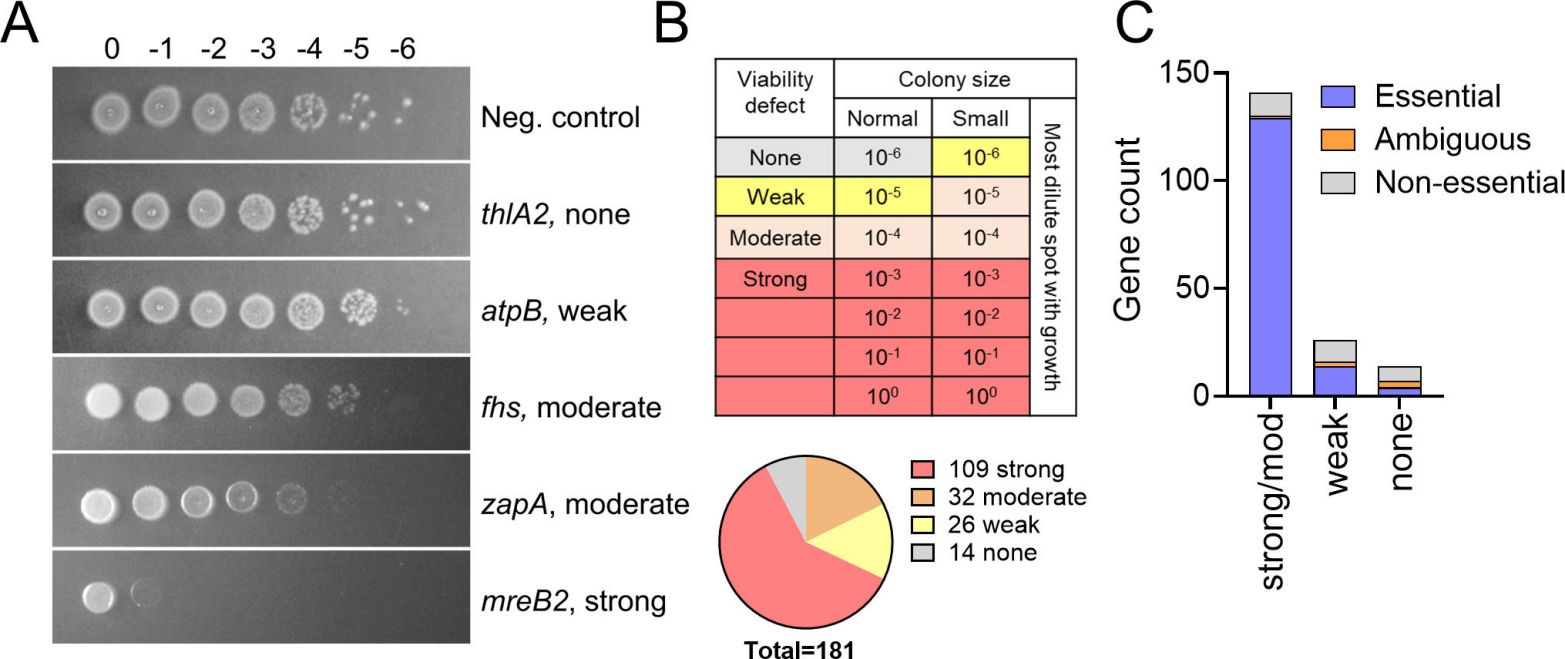
Summary of gene essentiality determined by CRISPRi. (**A**) Representative spot titer assays illustrating the range of viability defects observed. Strains of R20291 harboring CRISPRi plasmids were grown overnight in TY-Thi10 medium. Samples were serially diluted in 10-fold increments, and 5 µL of each dilution was spotted onto TY-Thi10 plates with 1% xylose to induce the expression of *dCas9*. Plates were photographed after incubation overnight. (**B**) Scoring of CRISPRi screen. Top: Criteria for assigning viability defects based on colony size and the highest dilution at which growth was observed. Bottom: 92% of genes tested showed a viability defect. In cases where the two sgRNAs produced different results, the stronger viability defect was used. (**C**) Viability defects in CRISPRi correlate with a likelihood that a gene will be scored as essential by Tn-seq. Viability defects are from Table S1. Tn-seq calls come from Table S3.

The entire CRISPRi library was screened for viability defects by conducting spot titer assays on TY plates containing thiamphenicol at 10 µg/mL (hereafter TY-Thi10) and 1% xylose. Control plates lacked xylose. Viability defects were scored as strong, moderate, weak, or none based on growth at different dilutions (Fig. 1A and B). Using a 10-fold viability defect or small colony phenotype with at least one sgRNA as the cut-off, 167 of the 181 genes (92%) were confirmed as essential by CRISPRi, while 14 were not essential (Fig. 1B; Table S1). Similar results were obtained with both sgRNAs for 174 of the 181 genes tested (Table S1). None of the 20 non-targeting control sgRNAs caused a growth defect, indicating off-target effects are rare. We conclude that the vast majority of the genes Dembek et al. identified as essential by Tn-seq are also essential by CRISPRi (14).

### Terminal phenotypes due to CRISPRi knockdown of genes of known function

To look for morphological abnormalities that might facilitate the provisional assignment of essential genes to functional pathways, cells were scraped from the last culture dilution that grew on the 1% xylose plates and examined by phase-contrast microscopy. As the project progressed, we added staining with FM4-64 to visualize the cytoplasmic membrane and Hoechst 33342 to visualize DNA. The morphological defects associated with CRISPRi silencing of all 181 genes are listed in Table S1.

**Fig 2:**
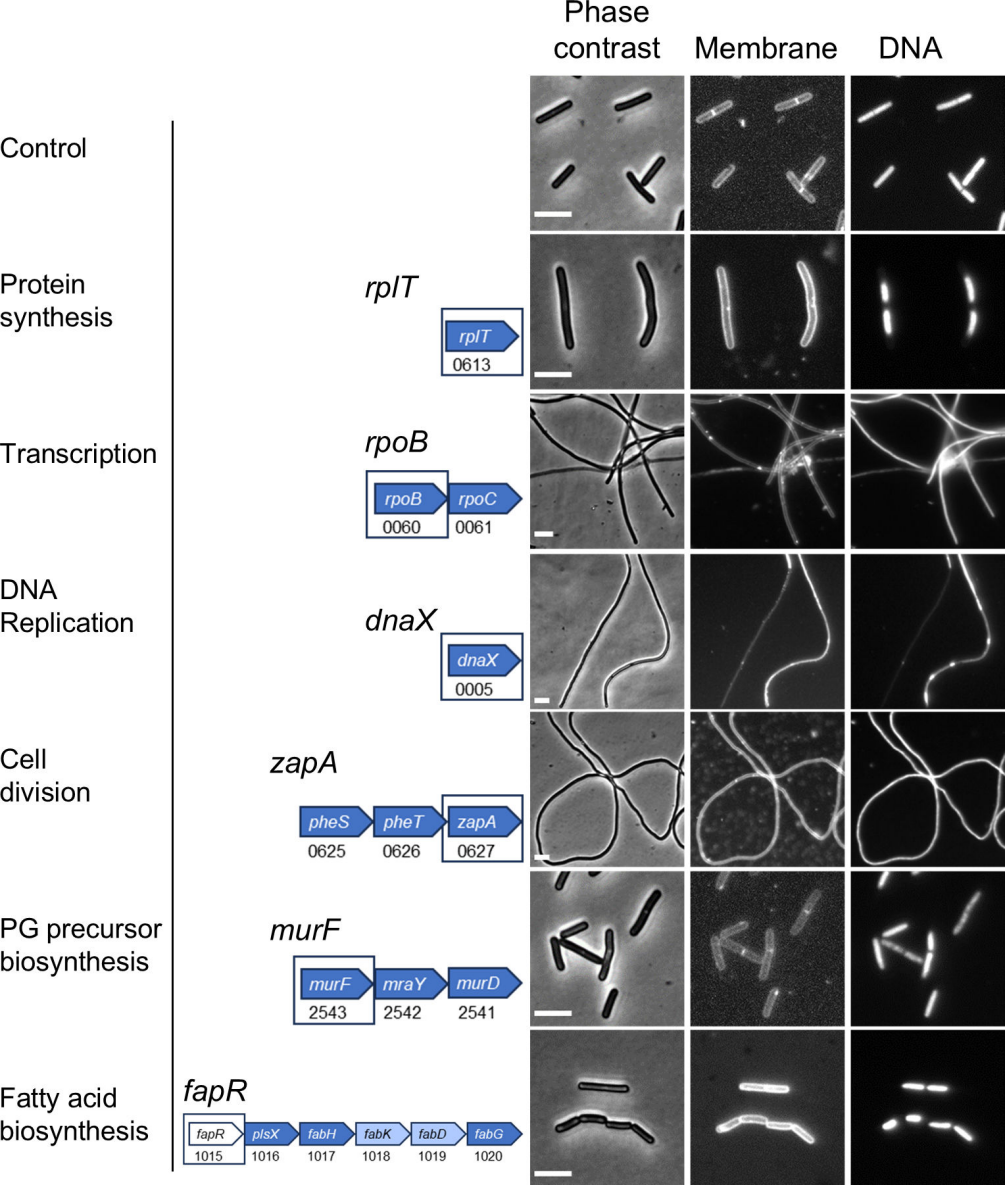
Morphology of CRISPRi strains with sgRNAs targeting genes in select functional pathways. Left: pathway. Middle: Predicted transcription unit. Targeted genes are boxed and indicated above the operon diagrams. Numbers are R20291 locus tags. Genes are color-coded to indicate essentiality based on Tn-seq calls in Table S3. Dark blue: essential. Light blue: ambiguous. White: non-essential. Operon structure is not to scale. Right: Morphological changes based on phase contrast and fluorescence micrographs of cells scraped from viability plates. Membranes were stained with FM4-64, and DNA was stained with Hoechst 33342. The size bars are 5 µm. The control strain expressed an sgRNA that does not target anywhere in the genome. Micrographs are representative of at least two experiments. Figure S1 shows microscopy of more genes.

CRISPRi knockdown of genes of known function often provoked expected morphological defects, such as filamentation in the case of cell division genes and aberrant nucleoid staining in the case of DNA replication genes (Fig. 2; Fig. S1, Table 1, Table S1). Also, as expected, knockdown of DNA replication genes sometimes resulted in filamentation, presumably due to induction of the SOS response (19, 20). However, we also observed morphological defects that were not expected and are difficult to rationalize. For instance, knockdown of *rpoB* (β subunit of RNA polymerase) or *era* (GTPase involved in ribosome assembly) caused severe filamentation, while knockdown of *guaA* (synthesis of guanosine ribonucleotides) caused a mild chaining phenotype. To address whether the unexpected morphological abnormalities are an artifact of working with cells scraped from plates, we reexamined the filamentation phenotype of four non-division genes in broth about six doublings after inducing CRISPRi: *dnaX*, *rpoB*, *prfB,* and *tilS*. We observed elongated cells in each case (Fig. S2). Thus, at least for this phenotype and these four genes, morphologies determined using plates are reliable.

**TABLE 1:** CRISPRi phenotypes of functional pathways

Pathway	Genes^*a*^	Phenotype^*b*^
Cell division	***ftsZ**, maf, **minC**, minD, whiA, **zapA***	Filaments, few septa, lysis, misshapen cells (swollen or bent), phase-bright cells, chaining, mostly normal chromosome morphology.
DNA replication	***dnaD**, dnaE, dnaF, dnaG, dnaL, **dnaX**, holA, priA, ssb*	Filamentous, few septa, condensed chromosomes, and regions devoid of DNA.
Fatty acid and phospholipid biosynthesis	*accB, acpS, cdsA, fabF, fabG2, **fabK**, fabZ, **fapR**, pgsA, plsC, plsY, yqhY*	Highly variable, including mostly normal morphology, short cells, elongated cells, misshapen cells (swollen or bent), phase-bright cells.
Nucleotides	***guaA**, guaB, nrdD, pyrH, thyA, tmk*	Mostly normal morphology, chaining, filamentous cells with areas of condensed chromosomes or devoid of DNA.
Peptidoglycan biosynthesis	***rodA**, **pbp1**, **pbp2**, murJ2*	pbp1: filamentous cells with few septa; pbp2 or rodA: short, swollen cells, often phase-bright, chaining. murJ2: misshapen cells (swollen or bent).
Peptidoglycan precursor biosynthesis	*ddl, glmU, mraY, murB, **murE**, **murF**, **murG**, murI*	Short cells, phase-bright cells, misshapen cells (swollen or bent), mild elongation, lysis.
Protein synthesis	*argS, cpgA, efp, **era**, fusA, infC, obg, prfA, prfB, rbgA, rimM, rlmL, rnrY, rplC, **rplT**, rpsM, **serS1**, smpB, thrS, tilS, tsf, tufA, **tufB***	Normal morphology, elongation, misshapen cells (swollen or bent), chaining, condensed chromosomes.
Secondary cell wall polymer PS-II	*cdr_2657, cdr_2663, **cdr_2665**, **gtaB**, murJ1, pgm2, rkpK, **tuaA**, tuaG*	Chains of short, swollen cells, sometimes phase-bright, sometimes mild elongation.
Transcription	***rpoA**, **rpoB***	rpoA: misshapen cells, a few modestly elongated; rpoB: long aseptate filaments, chromosome morphology normal.

^*a*^ Depletion phenotypes for genes in bold are shown in Fig. 2 and Fig. S1.

^*b*^ Phenotypes reported encompass the range observed across the genes listed. The phenotypic defects often differed for different genes from the same functional pathway. Major phenotypes caused by repression of each gene are listed in Table S1.

Because morphological defects were only loosely associated with the function of well-studied genes, we conclude that CRISPRi is not sufficient for assigning genes of unknown function to physiological pathways. We are not the first to report unanticipated complexity among terminal phenotypes in a CRISPRi screen. For example, CRISPRi knockdown of the RNA polymerase gene *rpoC* and the phospholipid synthesis genes *psd* and *plsB* caused filamentation in *E. coli* (8). In addition, knockdown of multiple genes with no direct role in envelope biogenesis caused morphological defects in *B. subtilis* (7). These reports contrast with the narrower spectrum of morphological defects induced by antibiotics that target specific pathways (21–23). Antibiotics might be less subject to secondary effects because cells are visualized at early times after exposure, and polarity is not an issue.

### Terminal phenotypes due to CRISPRi knockdown of genes of unknown function

Our CRISPRi library targeted 11 genes that could not be assigned to a functional category and were confirmed as essential in our own Tn-seq analysis, as will be described below. CRISPRi caused a viability defect in nine cases, often accompanied by abnormal morphologies (Table 2). Examples include *cdr20291_0481* and *cdr20291_0828* (elongation), the *cdr20291_1053-1057* cluster (short, swollen, phase-bright cells, and chaining), *cdr20291_1124* (chaining and many misshapen phase-bright cells), and *cdr20291_2526* (a few misshapen cells). The phenotype resulting from knockdown of *cdr20291_1124* could be due to reverse polarity onto the upstream gene *alaS*, which encodes an alanyl-tRNA synthetase. These genes warrant further investigation.

**TABLE 2:** Essential genes not assigned to a physiological pathway^*a*^

Locus tag	Annotation	Size	CRISPRi viability defect	CRISPRi terminal morphology
CDR20291_0351	Phosphoesterase	230 a.a.^*b*^	Weak	Normal
CDR20291_0481	Sugar isomerase/endonuclease	251 a.a.	Weak	Elongated
CDR20291_0828	DUF1846 domain	501 a.a.	Strong	Elongated
CDR20291_1053	Pyrophosphokinase	373 a.a.	Strong	Chaining, short cells, swollen cells, phase-bright
CDR20291_1054	Putative exported protein	291 a.a.	Strong	See CDR20291_1053
CDR20291_1055	Family 2 glycosyl transferase	230 a.a.	Strong	See CDR20291_1053
CDR20291_1056	Glycosyl transferase family protein	274 a.a.	Strong	See CDR20291_1053
CDR20291_1057	DUF3866 domain	355 a.a.	Not targeted	
CDR20291_1124	Putative membrane protein	723 a.a.	Moderate	Chaining, curved cells, phase-bright
CDR20291_1171	UvrD/REP type DNA helicase	593 a.a.	None	Normal
CDR20291_1418B	None	113 a.a.	Not targeted	Not done
CDR20291_2521	PDZ, radical SAM, and DUF512 domains	466 a.a.	Not targeted	Not done
CDR20291_2526	Two-component response regulator	230 a.a.	Moderate	Mostly normal, a few curved
CDR20291_2569	Putative calcium-chelating exported protein	308 a.a.	None	Normal
CDR20291_3525	Conserved hypothetical protein	61 a.a.	Not targeted	Not done

^*a*^ These proteins were classified as essential in our Tn-seq and either essential or ambiguous by Dembek et al., 2015. CDR20219_3519 and CDR20219_3520 are omitted because their essentiality is likely due to polarity onto *dnaC* and/or *rplI*.

^*b*^ a.a., amino acids.

### Rationale for the decision to conduct Tn-seq

As noted above, our CRISPRi screen was based on the only published genome-wide analysis of gene essentiality in *C. difficile*. That study made essentiality calls based on a single pool of mutants containing ~77,000 unique Tn insertions plated on BHIS media (14). We reasoned that an independently derived list of essential genes based on multiple biological replicates and larger insertion libraries would serve as a useful resource to the *C. difficile* community. We also thought a new Tn-seq data set might serve as a “tie-breaker” for the 14 putatively essential genes that did not appear to be essential by CRISPRi, that is, failure to recover insertions in those genes would suggest our sgRNAs were ineffective, while recovery of insertions would suggest the genes are non-essential and were missed in the previous study for stochastic reasons.

### Generation of Tn insertion libraries and identification of essential genes

We used the same R20291 strain and mariner-based transposon as in the previous study (14). Mariner is a good choice for *C. difficile* because it inserts at TA dinucleotides, and the genome G + C content is 29% (24, 25). However, our experimental design differed from Dembek et al. in several noteworthy respects: (i) we used TY media, (ii) we constructed three independent insertion libraries, (iii) we used a different algorithm for classifying genes as essential or non-essential, and (iv) we determined insertion profiles at both an early and a late timepoint because gradual loss of slow-growing mutants from the pools influences perceptions of gene essentiality. Our early timepoint consisted of primary insertion libraries recovered directly from selection plates after ~18 hours of incubation. For a later timepoint, libraries were sub-cultured in duplicate into TY and harvested after seven generations of outgrowth.

**Fig 3:**
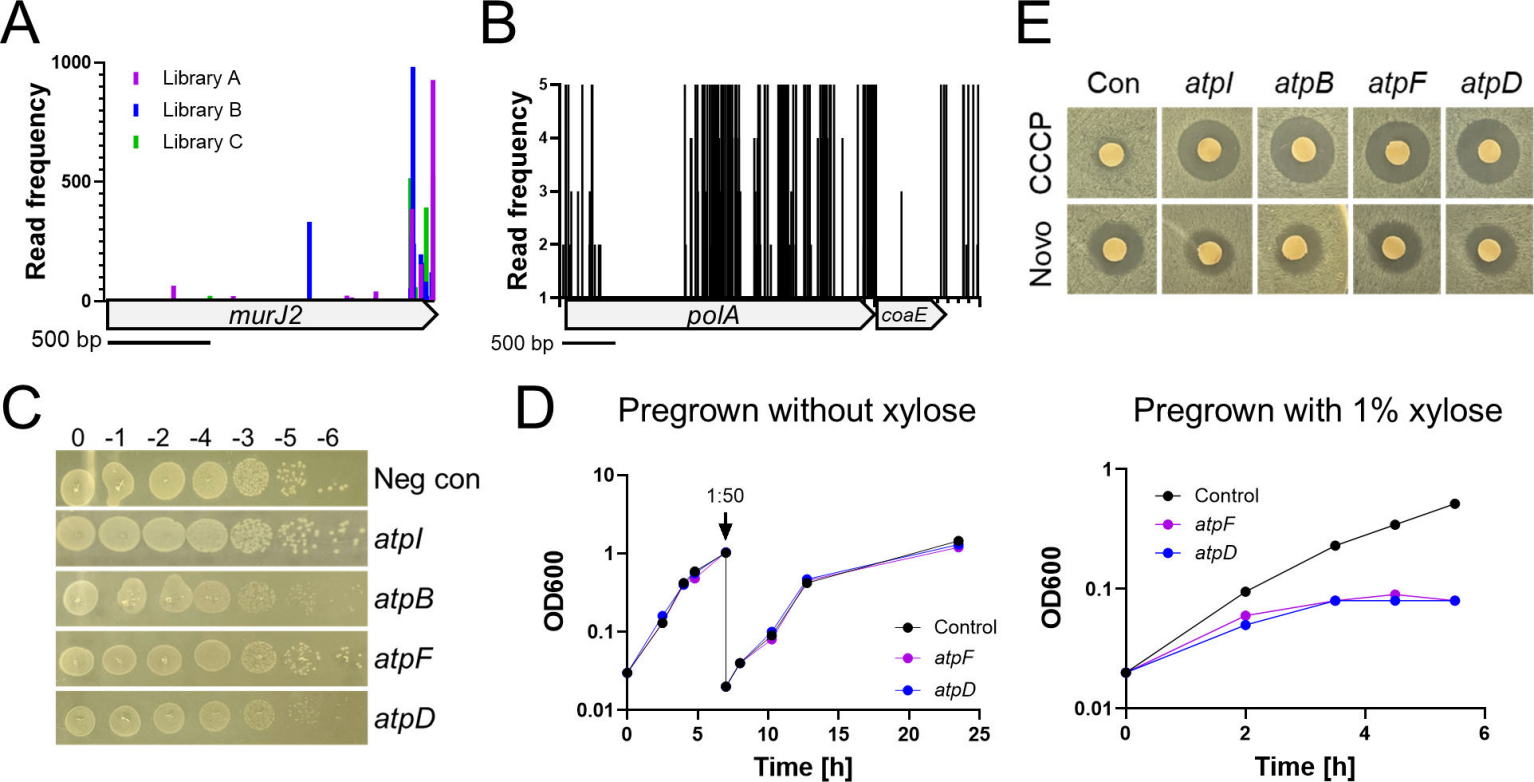
Essentiality follow-up. (**A**) Transposon insertion profile for *murJ2*. Vertical lines represent mapped insertion sites and are scaled to indicate the number of sequence reads mapping to that site. Although *murJ2* sustained numerous insertions, ~80% were in the last 10% of the gene, suggesting the non-essentiality call by TRANSIT2 is incorrect. (**B**) Transposon insertion profile for *polA* indicates that only the N-terminal domain is essential. Read frequency was scaled to 5 to highlight the absence of reads in the N-terminal domain. The average number of reads per *polA* site with at least one read was 173. (**C**) Spot titer assays of CRISPRi strains targeting genes in the *atp* operon. Serial dilutions of overnight cultures were spotted on TY-Thi10 plates with 1% xylose. Plates were imaged after incubation at 37°C for ~18 h. Silencing *atpB* and *atpD* resulted in small colonies, while growth after silencing *atpI* and *atpF* was comparable to the negative control. (**D**) Pre-depletion of ATP synthase proteins impairs growth. Starter cultures were grown overnight in TY-Thi10 without (left) or with (right) 1% xylose, then subcultured into TY-Thi10 with 1% xylose, and growth was followed by measuring optical density at 600 nm. To prolong growth, cultures in the left panel were back-diluted at 7 h. (**E**) Zone of inhibition assays reveal CRISPRi knockdown of the *atp* operon increases sensitivity to CCCP. Plates were imaged after incubation at 37°C for ~18 h. Novobiocin (Novo) served as a control. Guides in panels C–E were as follows: *atpI* (5531), *atpB* (5583), *atpF* (5581), *atpD* (5579), or a negative control that does not target anywhere in the genome.

Genomic DNA was extracted from libraries, and transposon insertion sites were identified by DNA sequencing following linear PCR, C-tailing, and the addition of barcodes and adaptors as described (26). This method was originally referred to as Tn-seq, and we have adopted that terminology here. Insertion profiles were analyzed using TRANSIT2 and the *C. difficile* R20291 reference genome NC_013316.1 (27, 28). Depending on the experimental replicate, insertions were identified in 117,217–204,061 of the 502,945 unique TA dinucleotides in the R20291 genome (Table 3). A total of 289,505 TA sites sustained at least one Tn insertion across the three libraries. TRANSIT2 makes essentiality calls by comparing the observed frequency of Tn insertions to the availability of potential TA insertion sites. Genes are classified as essential (E or EB, depending on the model for statistical analysis), not essential (NE), or unclear (U) (29). Genes with too few TA sites for statistical analysis are designated short (S). After inspecting the output from TRANSIT2, we manually reclassified 11 NE or U genes as essential, giving them the designation Ei for “essential by inspection.” Ten of these genes had a large number of TA sites but very few insertions. An example is the tRNA-synthetase *valS* (CDR20291_3114), with insertions in only four of the possible 266 TA dinucleotides after outgrowth (Table S3A). For comparison, TRANSIT2 scored the cell division gene *ftsZ* as essential even though there were insertions in 3 out of 110 TA sites. All 10 genes that we moved to Ei based on a few insertions are considered essential in *C. difficile* and *B. subtilis* (14, 30). The final Ei gene, *murJ2* (CDR20291_3335), had a large number of insertions, but almost all of these were at the 3′ end of the gene, suggesting a functional protein is still produced (Fig. 3A). *murJ2* was previously classified as essential in *C. difficile* by Dembek et al., but its ortholog is not essential in *B. subtilis* due to functional redundancy (30, 31).

**TABLE 3:** Number of unique transposon insertions from experimental replicates^*a*^

Replicate	Unique TA sites hit	Fraction of total TA sites
Library A	178,325	0.355
Library B	168,519	0.335
Library C	143,213	0.285
Combined Libraries A–C	289,505	0.576
Outgrowth A1	135,217	0.269
Outgrowth A2	117,217	0.233
Outgrowth B1	127,947	0.254
Outgrowth B2	135,056	0.269
Outgrowth C1	167,894	0.334
Outgrowth C2	204,061	0.406

^*a*^ Total TA sites = 502,945.

Of the 3,673 annotated protein-coding genes in R20291, 346 were scored as essential for vegetative growth in the initial libraries and/or after outgrowth (Table S3A). We grouped these genes into functional categories similar to those used in previous studies of *B. subtilis* and *S. aureus* (Table 4 and Table S3B) (32, 33). As expected, over half are involved in DNA metabolism (25 genes), RNA metabolism (24 genes), protein synthesis (113 genes), or cell envelope biogenesis (76 genes). Also, as expected, the majority of *C. difficile’s* essential genes are conserved; BioCyc assigned a *B. subtilis* ortholog for 272 of the 346 genes, of which 169 are essential (Table S3A and B) (30).

**TABLE 4:** Tn-seq essential genes by category

Category	Count
Cell envelope	**76^*a*^**
Cell division	7
Cell shape	4
Diaminopimelate biosynthesis	7
Fatty acid biosynthesis	7
Isoprenoid biosynthesis	7
Peptidoglycan biosynthesis	5
Peptidoglycan precursor biosynthesis	13
Phospholipid biosynthesis	6
Regulation	3
S-layer	2
Secondary cell wall polymer PS-II	14
Other	1
Cofactors	**24**
CoA	6
Fe-S cluster	1
Folate	7
Heme	1
NAD	4
Riboflavin	1
SAM	1
Thiamine	3
DNA metabolism	**25**
DNA packaging and segregation	4
DNA recombination and repair	4
DNA replication	16
Other	1
Metabolism	**15**
Amino acid biosynthesis	3
Glycolysis	2
Pentose phosphate pathway	1
Phosphate metabolism	1
Other	8
Nucleotides	**11**
dNTP biosynthesis	2
Purine biosynthesis	3
Pyrimidine biosynthesis	4
Regulatory nucleotides	2
Other/unknown	**58**
Phage-related	4
Sporulation	2
Transporter	6
Transposon-related	17
Other	1
Unknown	28
Protein synthesis	**113**
Protein degradation	4
Protein folding	2
Protein modification	2
Protein translocation	7
Ribosomal proteins	52
Ribosome biogenesis	12
Translation factors	10
tRNA synthetases	24
RNA metabolism	**24**
Basic transcription machinery	5
Regulation of RNA synthesis	4
RNA processing and degradation	6
tRNA modification	9
Total	**346**

^*a*^ Values in bold represent the total count for each category.

The above numbers are derived from analyzing all experimental replicates together, raising questions about how having three independent libraries and an outgrowth step influenced essentiality calls. TRANSIT2 flagged 283, 370, and 328 genes as essential when the libraries were analyzed individually, but that number dropped to 263 in the combined analysis (Table S3C). Of these 263 genes, 219 were essential in all three libraries, 19 in two libraries, and 15 in only one library. Ten genes were classified as essential in the combined analysis, even though they were not essential in any individual library. Thus, overall, there was good agreement across the three primary insertion libraries, and the primary effect of combining three libraries was to reduce the number of genes classified as essential. This makes biological sense because as the number of Tn insertions goes up, the number of non-essential genes that lack insertions for stochastic reasons goes down. Conversely, 72 genes were newly classified as essential based on the outgrowth experiments, bringing the total to 335 (263 + 72 = 335; Table S3A). This increase makes biological sense because mutants are lost from the pool during outgrowth for two reasons: bottlenecks at subculturing (a source of false positives) and gradual loss of slow-growing mutants from the pool (genes that are truly essential or quasi-essential). Inclusion of 11 genes that were classified as essential by inspection brings the final overall tally to 346.

### Comparison of our Tn-seq data to Dembek et al.

**Fig 4:**
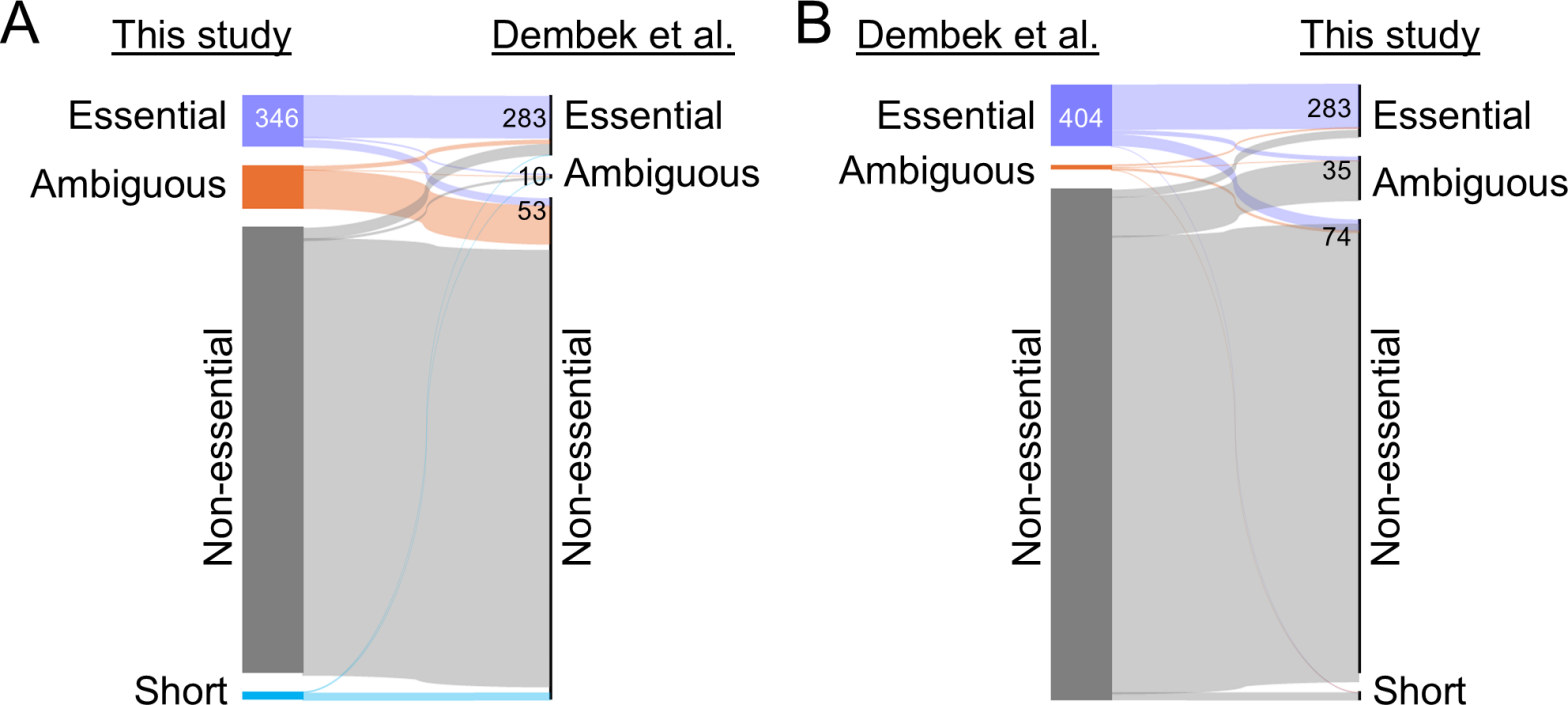
Comparison of Tn-seq data sets using Sankey diagrams. (**A**) Of the 346 genes classified as essential in this study, 283 were essential, 10 were ambiguous, and 53 were non-essential for Dembek et al. (**B**) Of the 404 genes classified as essential by Dembek et al., 283 were essential, 35 were ambiguous, and 74 were non-essential in this study. The thickness of each color line is proportional to the number of genes, and labels on the left and right indicate essentiality calls. Short refers to genes that are too short to be called by TRANSIT2. Note that 12 genes identified as essential by Dembek could not be mapped to our data set as they were not present in the genome annotation used here. Category “ambiguous” combines unclear with unclear/NE from Table S3.

There is good overall agreement between our Tn-seq essentiality calls and those made previously. Of the 346 genes identified as essential in our experiments, 283 (82%) were also essential for Dembek et al. (Fig. 4; Table S3A). Overall, however, we scored fewer genes as essential, 346 versus 404 (Fig. 4). This is a difference of 58 genes and comprises 121 genes uniquely essential for Dembek et al. and 63 genes uniquely essential for us (404 − 121 + 63 = 346). These differences could reflect both differences in experimental design and the randomness inherent in transposon insertion sequencing, and both data sets are expected to include some misclassified genes. Distinguishing between these sources of variation in the case of specific genes would require follow-up experiments, which we did not undertake. Nevertheless, we can speculate. In particular, we suspect the primary reason we identified fewer genes as essential is that our insertion libraries were much larger. If the primary driver were the use of different media, we would expect discrepancies to be skewed toward more essentiality of metabolism genes. Of the 63 genes uniquely essential to our study, only 14 appear to be directly involved in metabolism (*brnQ1*, *ctfA*, *cmk*, *dpaL, fchA*, *gutB*, *hisC*, *ilvB*, *ilvC*, *panB*, *pspA*, *rpe*, *thiG,* and *yidE*). If the primary driver of differences were our reanalysis of the mutant pools after outgrowth, we should have classified more genes as essential than did Dembek et al., but the opposite is true. Moreover, of the 72 genes that made our list because they became essential during outgrowth, only 25 were non-essential in Dembek et al., and inspection of this gene list does not reveal any obvious reasons why these 25 genes would be uniquely important during prolonged growth.

A further potential source of differences is the use of different algorithms for making essentiality calls. Naively, one might suppose that the presence of even a single Tn insertion in a gene would be sufficient to score that gene as non-essential, in which case the algorithm should not matter. But, in practice, insertions can be mapped to genes by errors in work-up steps. In addition, some regions of the chromosome are more accessible to transposons than others. Lastly, insertions into the 3′ end of a gene might not inactivate it. For these and other considerations, a variety of algorithms have been developed for making essentiality calls based on the availability of TA sites and the local density of insertions observed. We do not know to what extent use of different algorithms explains differences in essentiality calls between the two *C. difficile* studies, but one potential example is the DNA polymerase gene *polA*, which was classified as essential by us but not by Dembek et al. Although *polA* sustained Tn insertions at 205 of its 312 TA sites in our experiments, these insertions were restricted to the 3′ end of the gene, implying *polA* has an essential N-terminal domain (Fig. 3B; discussed below).

### Comparison of our Tn-seq data to results obtained by CRISPRi

There is good overall agreement between our CRISPRi and Tn-seq data sets. Of the 141 genes for which CRISPRi elicited a strong or moderate viability defect, 129 (~90%) scored as essential in our Tn-seq (Fig. 1C; Table S3A). Conversely, only 4 out of 14 genes (~30%) that appeared to be non-essential by CRISPRi nevertheless scored as essential in our Tn-seq. These four genes are an uncharacterized DNA helicase (CDR20291_1171), a sporulation-associated phosphatase (*ptpB*), an acetyl-CoA thiolase (*thlA2*), and a putative exported Ca^2+^-chelating protein (*ykwD*). None of these has an essential ortholog in *B. subtilis*. Two labs have constructed null mutants of *ptpB*, indicating it is not essential (34, 35). One study reported a growth defect (35), which might explain why *ptpB* appears to be essential by Tn-seq.

### DNA metabolism

Some DNA replication proteins have different names in *B. subtilis* and *E. coli*. Where there are conflicts, we adopted the names used in *B. subtilis*, which, in some cases, differ from the names used in BioCyc. We identified 16 widely conserved DNA replication genes as essential in *C. difficile*. All but *pcrA* and *polA* were previously classified as essential in *C. difficile*, and all but *polA* are essential in *B. subtilis* (14, 30). Interestingly, *polA* is domain essential in *C. difficile*—Tn insertions were recovered in the C-terminal 3′ to 5′ exonuclease and DNA polymerase domains but not in the N-terminal 5′ to 3′ exonuclease domain, which removes Okazaki fragments (Fig. 3B). Similar restricted essentiality of the *polA* 5′ to 3′ exonuclease domain has been reported in *Streptococcus* and *Haemophilus* (36, 37). Organisms like *B. subtilis,* in which the entire *polA* gene is dispensable, have an RNAse H that can remove Okazaki fragments (38).

Interestingly, *C. difficile* lacks *dnaB* (39). DnaB is an essential protein in *B. subtilis*, where it works together with DnaD and DnaI to load the replicative helicase DnaC onto *oriC* DNA (40). DnaB and DnaD are structurally related. It has been proposed that in *C. difficile,* the DnaD ortholog (CDR20291_3512) fulfills the functions of both DnaB and DnaD (39).

*C. difficile* has four essential DNA packaging and segregation genes, all of which are also essential in *B. subtilis*. In addition, there are three essential DNA recombination and repair genes, none of which are essential in *B. subtilis*.

LexA, which represses genes involved in the SOS response, is required for viability in *C. difficile* but not in *B. subtilis*. A *C. difficile lexA* Clostron insertion mutant has been described and grows poorly, so its apparent essentiality by Tn-seq may be due to slow growth rather than lack of viability *per se* (19). However, the strong viability defect we observed upon CRISPRi knockdown of *lexA* (Table S1) raises the possibility that the reported mutant retains partial function or acquired a suppressor.

### RNA metabolism

As expected, the core subunits and major sigma factor (σ^70^) of RNA polymerase are all essential. Surprisingly, the omega subunit (*rpoZ*) is also essential according to Tn-seq, even though it is not essential in *B. subtilis*, *S. aureus,* or *E. coli* (30, 41, 42). The apparent essentiality of *rpoZ* is likely to be an artifact of polarity because it is predicted to be co-transcribed with three widely conserved essential genes: *dapF*, *gmk*, and *coaBC*. The elongation factor *greA* and three termination/anti-termination factors (*nusA*, *nusG,* and *rho*) are essential. Of these, only *nusA* is essential in *B. subtilis* (30, 43). In *C. difficile, rho* mutations have been reported, including an early frameshift, but the gene could not be deleted, possibly because the mutant is too sick (44).

In all, 15 genes for enzymes that modify RNA were essential in our analysis, of which 12 were essential or ambiguous for Dembek et al., but only 8 are considered essential in *B. subtilis*. Most of these genes encode proteins needed to generate mature tRNAs or rRNAs from precursor transcripts.

### Protein synthesis

There are 55 annotated *C. difficile* ribosomal proteins in BioCyc (Dec 18, 2024), 52 of which scored as essential by our Tn-seq. Most of these were also identified as essential by Dembek et al. and are essential in *B. subtilis*. Instances of ribosomal protein genes scored as essential in *C. difficile* but as non-essential in *B. subtilis* could reflect polarity. Five widely conserved small GTPases involved in ribosome assembly are essential, as are 10 translation factors, including *smpB*, which encodes a component of the SsrA tagging complex that rescues stalled ribosomes by trans-translation (45). We confirmed the essentiality of *smpB* by CRISPRi (Table S1; “weak” viability defect). The essentiality of *smpB* is unlikely to be an artifact of polarity because it is not predicted to be co-transcribed with any other genes. SmpB is essential in *S. aureus* (33, 46) but not in *E. coli*, *Streptococcus sanguinis*, or *B. subtilis* (30, 47, 48). An interesting omission from the list of essential translation factors is elongation factor Tu (EF-Tu), which is essential in *B. subtilis* (30). This difference can be explained by the presence of two EF-Tu genes in *C. difficile*, *tufA* and *tufB*, which are 100% identical at the DNA level. Simultaneous knockdown of *tufA* and *tufB* with CRISPRi caused a strong viability defect, demonstrating EF-Tu is indeed required for viability (Table S1).

We identified 24 essential tRNA synthetases, all of which are also essential according to Dembek et al. There are several noteworthy differences in comparison to *B. subtilis*. First, synthetases for asparagine (*asnS*), threonine (*thrS*), and tyrosine (*tyrS*) are essential in *C. difficile* but not *B. subtilis*, which has alternative routes for generating the corresponding charged tRNAs (49–51). Second, although *glnS* is essential in *C. difficile*, this gene does not exist in *B. subtilis* or most other Gram-positive bacteria, which generate Gln-tRNA^Gln^ by a different route. Namely, *C. difficile* charges tRNA^Gln^ directly with glutamine, as in *E. coli*, while most Gram-positive bacteria generate glutaminyl-tRNA^Gln^ by (mis)charging tRNA^Gln^ with glutamate, which is then amidated to glutamine (52, 53). Lastly, *C. difficile* has two annotated genes for ligating proline to tRNA^pro^, the essential gene *proS1* (CDR20291_0038) and the non-essential gene *proS2* (CDR20291_0039). According to RNA sequencing, both are expressed during vegetative growth (54). *B. subtilis* has only a single *proS* gene, which is essential and more similar to *C. difficile proS1* than *proS2*.

Five proteases appear to be important for viability in *C. difficile: clpX*, *htrA*, *lon, prp,* and the M16 family protease *cdr20291_1161*. Of these, only *prp* is essential in *B. subtilis*. Prp is a cysteine protease needed to remove an N-terminal extension from ribosomal protein L27 (55). The apparent essentiality of *lon* and *cdr20291_1161* in *C. difficile* is likely to be an artifact of polarity onto *engB* and *dapG*, respectively. ClpX is a component of the ClpXP protease complex, one of the major housekeeping proteases in bacteria (56). *C. difficile* has only one *clpX* gene but two genes for ClpP, which might explain why *clpX* is essential but *clpP1* and *clpP2* are not. HtrA proteases are involved in protein quality control (57). TRANSIT2 scored *htrA* as essential despite a high number of Tn insertions (67 out of 127 TA sites), and this gene was not essential for Dembek et al. Moreover, *htrA* has been inactivated in strain 630Δ*erm*, further indicating it is not truly essential (58).

In bacteria, protein synthesis begins with *N*-formyl methionine (fMet). Peptide deformylase (*def*) and methionine aminopeptidase (*map*) are essential enzymes that work sequentially to remove the formyl group from about 90% of proteins and the initiating methionine from about half of proteins. *E. coli* has only one *def* and one *map* gene, both of which are essential (59). *C. difficile* has two predicted *map* genes and two predicted *def* genes. Of these, only *map1* is essential by Tn-seq. This situation is reminiscent of *B. subtilis*, which also has two *def* and two *map* genes. The *def* genes are functionally redundant and at least one must be present for viability (60, 61). The essentiality of the *map* genes *in B. subtilis* is less clear. One study found *mapA* is essential, but *mapB* is not (62), while another found neither is individually essential (30).

Bacteria have a plethora of systems for exporting proteins out of the cytoplasm, of which the three most important are the General Secretion (Sec) system, the Twin Arginine Translocation (Tat) system, and the Signal Recognition Particle (SRP) system (63). There is no Tat system in *C. difficile*, but the genes for the Sec and SRP systems are present and essential. The Sec system uses an ATPase named SecA to power the export of proteins through a membrane channel composed of SecEYG. Interestingly, *C. difficile* has two *secA* paralogs, which handle different protein substrates and are both essential (64). The SRP system works together with SecEYG to integrate proteins into the cytoplasmic membrane. Three genes associated with the SRP system (*ffh*, *ftsY,* and *srpM*) were scored as essential, although the apparent essentiality of *srpM* might result from polarity onto *ffh; srpM* is not essential in *B. subtilis*.

### Cell envelope

Numerous genes involved in membrane biogenesis are essential in *C. difficile*. An unexpected exception is the *accBCDA* gene cluster for the synthesis of malonyl-CoA, the substrate for fatty acid synthesis. This result is difficult to explain and probably incorrect because the *acc* cluster is essential according to Dembek et al., and we confirmed essentiality by CRISPRi (Table S1). Moreover, *acc* genes are also essential in *B. subtilis* (30). Nevertheless, the *acc* cluster sustained numerous Tn insertions in our study (e.g., 10 of the 48 TA sites in *accB*, the first gene in the operon). We identified three membrane biogenesis genes that are essential in *C. difficile* but not in *B. subtilis: fabH*, *yqhY*, and *gpsA*. The *fabH* discrepancy can be explained by the presence of two *fabH* genes in *B. subtilis* (65). *B. subtilis* Δ*yqhY* mutants are not stable (66), implying *yqhY* is quasi-essential in that organism. Regarding *gpsA*, although Koo et al. reported it is dispensable in *B. subtilis* (30), an earlier study found it is essential (67), which agrees with what we see in *C. difficile*.

*C. difficile* synthesizes isoprenoids via the methylerythritol (MEP) pathway (68). Accordingly, *dxr* and *ispDEFGH* were all essential by Tn-seq. Isoprenoids are essential in bacteria because they are precursors for quinones and carrier lipids, such as undecaprenyl phosphate (Und-P) required for the synthesis of peptidoglycan and teichoic acids (69). *C. difficile* lacks quinones (70), so the essentiality of the MEP pathway presumably reflects the importance of Und-P. Consistent with this inference, the predicted undecaprenyl pyrophosphate synthase UppS1 is essential, although that conclusion comes with a caveat because insertions in *uppS1* are probably polar onto the essential phospholipid biosynthesis gene *cdsA* (71). Interestingly, *C. difficile* has a non-essential *uppS* paralog called *uppS2* that might be involved in the synthesis of a secondary cell wall polymer called PS-II (72). UppS2 is not essential by Tn-seq, and RNA sequencing implies expression of *uppS2* is ~60-fold lower in vegetative cells compared to *uppS1* (54).

The *C. difficile* cell has a unique proteinaceous surface layer (S-layer) and, as mentioned above, a unique wall polymer called PS-II, which is thought to function like teichoic acids found in Gram-positive model organisms but whose structure is quite different (73). Both the S-layer and PS-II are essential by Tn-seq, although the existence of (unhealthy) null mutants of *slpA* indicates the S-layer is not strictly required for viability (74, 75). Multiple studies point to the essentiality of PS-II (72, 76, 77). Whether PS-II is essential because it plays a critical role in cell envelope integrity or because disruption of the PS-II gene cluster depletes the pool of Und-P needed for peptidoglycan synthesis remains to be determined (78, 79).

The universal precursor for peptidoglycan synthesis is lipid II, a disaccharide-pentapeptide attached to Und-P (80). As expected, many lipid II genes are essential, including six *dap* genes for biosynthesis of lysine and diaminopimelic acid, and nine *mur* genes for various steps in lipid II assembly. Lipid II is transported across the cytoplasmic membrane by flippases, of which there are two known families, MurJ and Amj (31, 81). BLAST searches indicate that *C. difficile* lacks Amj but has two MurJ homologs, both of which are essential. MurJ1 is part of the PS-II gene cluster and is proposed to transport a lipid-linked precursor for PS-II synthesis (76), which leaves MurJ2 as the likely lipid II flippase for peptidoglycan synthesis. Some non-essential proteins distantly related to MurJ can be identified using HHPred and could also potentially transport lipid II (82, 83). Further work is needed to establish the functions of the two clear MurJ homologs and rule out the presence of alternative or additional lipid II transporters (31, 84, 85).

**Fig 5:**
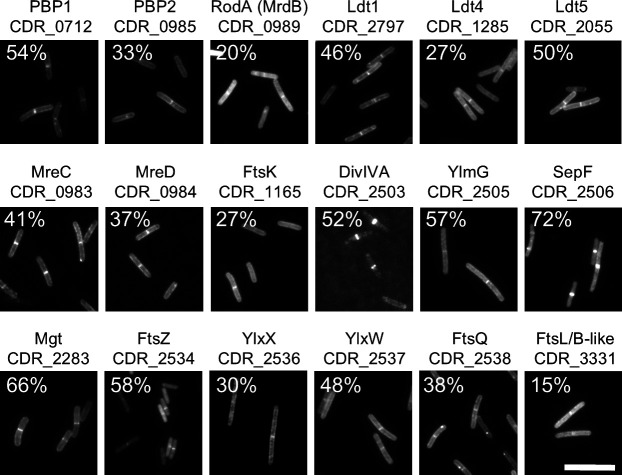
Representative fluorescence micrographs of fixed cells that produced the indicated proteins fused to RFP. Percentages indicate the fraction of cells scored positive for septal localization (*n* ≥ 202 cells). Size bar = 10 µm.

The final steps of peptidoglycan synthesis involve incorporation of new disaccharide-pentapeptide subunits into the existing wall by sequential glycosyltransferase (GTase) and transpeptidase (TPase) reactions (86, 87). These reactions are catalyzed by two types of penicillin-binding proteins (PBPs) (88). Class A PBPs (aPBPs) are bifunctional enzymes with both a GTase domain and a TPase domain, while class B PBPs (bPBPs) have a TPase domain and form a complex with a SEDS-family GTase (89–91). *C. difficile* encodes one aPBP (PBP1), three bPBPs (PBP2, PBP3, and SpoVD), and two SEDS proteins (RodA and SpoVE). Of these proteins, we confirmed by Tn-seq that PBP1, PBP2, and RodA are essential for vegetative growth (14). Although *spoVE* was also classified as essential, it sustained Tn insertions in about half the available TA sites (Table S3A), and the gene has been deleted previously (92). In confirmation and extension of previous reports (15, 93), CRISPRi knockdown of PBP1 caused filamentation, while CRISPRi knockdown of PBP2 and RodA resulted in the formation of short, swollen, phase-bright cells, with some chaining (Fig. S1). These morphologies implicate PBP1 in cell division and PBP2 in elongation, respectively. We also examined red fluorescent protein (RFP) fusions to the PBPs and observed that both localize to division sites (Fig. 5). Septal localization of PBP1 has been reported by Shen’s group, who showed that it is the primary synthase for septal peptidoglycan (93). Septal localization of PBP2 suggests the RodA/PBP2 complex might also contribute to cell division, as further suggested by the mild chaining phenotypes caused by CRISPRi knockdown. Both RFP-PBP1 and RFP-PBP2 exhibited some fluorescence along the cell cylinder, which could indicate they contribute to elongation, especially in the case of PBP2. However, localization to the cell cylinder is not diagnostic of a function in elongation because this is the default location of divisome proteins when they are not at the septum. Finally, it should be noted that non-canonical 3-3 crosslinks made by L,D-transpeptidases (LDTs) are essential for vegetative growth in *C. difficile*, but none of the five LDTs in the *C. difficile* genome is individually essential owing to functional redundancy (94).

Our Tn-seq identified two cell envelope-related regulatory loci as essential: *walRK* and *ddlR*. These regulators were also essential for Dembek et al. *walRK* is a two-component system known to be essential for cell wall homeostasis and viability in numerous Bacillota, including *C. difficile* (54, 95). DdlR is essential for peptidoglycan synthesis because it activates expression of the D-alanyl-D-alanine ligase *ddl* (96).

### Cell shape and division

In rod-shaped bacteria, the essential peptidoglycan synthases work in the context of loosely defined complexes known as the elongasome and the divisome (86, 87). The *C. difficile* elongasome appears to comprise the RodA/PBP2 bipartite peptidoglycan synthase and four Mre proteins (MreB1, MreB2, MreC, and MreD). All of these are essential by Tn-seq and CRISPRi, although this inference will need to be revisited with non-polar deletions. CRISPRi knockdown implicates these genes primarily in elongation, because the predominant terminal morphologies include short, swollen cells with some chaining (Fig. S1; Table S1).

Among canonical divisome proteins, only *ftsZ* and its assembly factors *sepF* and *zapA* are essential in *C. difficile*. Neither *sepF* nor *zapA* is essential in *B. subtilis* (97–99). The greater importance of *sepF* and *zapA* in *C. difficile* might be due to the absence of an *ftsA* ortholog (100). As noted above, the primary septal peptidoglycan synthase is the class A enzyme PBP1 (93). Consistent with that inference, CRISPRi against *pbp1* induces filamentation; however, additional morphological defects such as bending and chaining suggest PBP1 might contribute to elongation as well (15). Curiously, the division site placement genes *minCDE* are essential in *C. difficile*. This result might be an artifact of polarity onto the essential SEDS gene *rodA*, because the Min system is not essential in *B. subtilis* or most other bacteria (101). Tn-seq identified *maf* as essential. Maf is a nucleotide pyrophosphatase whose overproduction causes filamentation in both *B. subtilis* and *E. coli*, but Maf is not essential in either organism (102–104). The DNA-binding protein WhiA was essential for Dembek et al., and we observed a weak viability defect and modest cell elongation by CRISPRi, but *whiA* is not essential in our Tn-seq experiments. WhiA is conserved in monoderms and essential in *Mycobacterium tuberculosis* but not *Streptomyces* or *B. subtilis*, where it has been linked to cell division and chromosome segregation (105–109).

### Use of RFP fusions to identify new divisome proteins

We have a long-standing interest in bacterial cell division, so we extended our studies to include a screen for divisome proteins (110–114). Using CRISPRi knockdown to identify divisome proteins by screening for a filamentous phenotype comes with two major caveats—polarity onto a *bona fide* division gene will generate false positives, and depletion of non-essential divisome proteins might not cause cells to become longer than normal. A more direct approach is to use fluorescent tags to screen for proteins that localize to the division site. Here, the major caveat is that the tag might interfere with proper localization. We used BLAST searches to identify homologs of known morphogenesis proteins, which were fused to a codon-optimized RFP and produced from a plasmid under control of the xylose-inducible promoter, P_xyl_ (15). Some of these proteins are encoded in (predicted) operons with proteins of unknown function, so we constructed RFP fusions to several of these as well. Although septal localization is strong evidence for a role in cell division, the lack of septal localization is uninformative because we did not test whether our RFP fusions are functional. We screened a total of 25 proteins, of which 18 localized and are discussed below (Fig. 5). The seven that did not localize are MreB1, MreB2, FtsL, FtsB, SpoVE, CDR_3330, and CDR_2504.

Seven enzymes for peptidoglycan synthesis exhibited convincing midcell localization, including the two essential PBPs (PBP1 and PBP2), one essential SEDS protein (RodA), one non-essential monofunctional glycosyltransferase related to PBPs (Mgt), and three non-essential LDTs (Ldt1, Ldt4, and Ldt5). Of these, PBP1 was already known to localize to sites of cell division (93), but septal localization of the remaining enzymes is new and suggests they too contribute to the synthesis of septal peptidoglycan. Somewhat surprisingly, the canonical elongasome proteins MreC and MreD localized strongly to the midcell, even though our fusions to MreB1 and MreB2 did not. Mre proteins have been reported to localize transiently at or near the midcell in a few other bacteria (115–118). Further work is warranted to investigate the role of the Mre proteins in *C. difficile* and the possibility that MreC and MreD localize independently of MreB, for which there is precedent from non-rod-shaped bacteria that have MreC and MreD but lack MreB (118, 119).

*C. difficile* orthologs of five widely conserved divisome proteins localized to the midcell: FtsZ, FtsK, FtsQ, SepF, DivIVA, as did CDR_3331, a unique protein with limited structural similarity to both FtsL and FtsB, which in *C. difficile* are used for asymmetric division during sporulation (14, 93). Septal localization of *C. difficile* FtsZ has been reported previously (120). Septal localization of FtsQ is new but probably misleading because *C. difficile ftsQ* is a sporulation gene and not expressed during vegetative growth (14, 54, 92), whereas we produced RFP-FtsQ from P_xyl_. Immediately downstream of *ftsQ* are two genes of unknown function, *ylxW* and *ylxX*, that, according to RNA sequencing, are expressed in vegetative cells (54). YlxW and YlxX are encoded downstream of *ftsQ* in many Bacillota and have been proposed on this basis to play a role in envelope biogenesis (121). Our observation that these proteins localize to the midcell argues that they are involved in cell division. Another novel divisome protein identified in our screen is YlmG, a small membrane protein encoded in the *sepF* operon of many Gram-positive bacteria and Cyanobacteria (100). Mutants of *ylmG* have been constructed in several organisms and exhibit thin septa, poor sporulation, and/or aberrant nucleoid compaction and segregation, depending on the species (100). In closing, and for completeness, we note that four additional proteins have been shown previously to localize to the division site in *C. difficile*: ZapA, MldA, MldB, and MldC (114, 122). This brings the total number of documented divisome proteins to 22.

### Metabolism

For an insightful overview of energy metabolism in *C. difficile*, readers are referred to a review by Neumann-Schaal et al. (123). Briefly, *C. difficile* is an obligate anaerobe that generates energy through fermentation of sugars and amino acids, the latter by a process known as Stickland reactions (124, 125). There is no electron transport chain. Hence, the five genes that are essential for menaquinone biosynthesis in *B. subtilis* are not found in *C. difficile’s* genome. The TCA cycle is incomplete and is used to generate precursor metabolites rather than energy. Fermentation pathways generate ATP directly by substrate-level phosphorylation but can also be used via electron bifurcation and the Rnf complex to generate a motive force across the cytoplasmic membrane (126, 127). Whether this is a proton or a sodium-ion motive force is not yet known; we will assume protons for simplicity, that is, a PMF*. C. difficile* has an F_0_F_1_-type ATP synthase, which, depending on the needs of the organism, can consume the PMF to generate ATP or hydrolyze ATP to generate a PMF.

Not very many of the genes involved in these various pathways scored as essential by Tn-seq. Genes for the TCA cycle, acetate kinase, and the major Stickland reductases for glycine, proline, and leucine are all non-essential, as are the genes for the RNF complex and three electron bifurcation complexes (*etf* genes). The essentiality of genes for glycolysis is less clear because eight of these were essential for Dembek et al., but only two (*eno*, *tpiA*) were essential in our experiments. Glycolysis might have more of a contribution to growth on BHIS, which contains glucose, than to growth on TY. Differences in slow growth and statistical cutoffs that impact essentiality calls may also factor into the discrepancies. In support of this explanation, we observed a small colony phenotype when we used CRISPRi to knock down expression of four glycolysis genes (*fba*, *gapB*, *pgi*, and *pfkA*) that were essential for Dembek et al. but not in our Tn-seq (Table S1). A further point to keep in mind is that glycolysis genes could be more important for supplying precursor metabolites rather than energy in *C. difficile*.

A noteworthy discrepancy concerns the 10-gene operon for the F-type ATPase. Dembek et al. scored 9 of the genes as essential, but all 10 were non-essential in our Tn-seq experiments. This gene cluster is too large to have escaped Tn insertions by chance. The most likely explanation for this discrepancy has to do with how slow growth affects perceptions of essentiality. In support of this interpretation, we observed that CRISPRi knockdown of *atpB* and *atpD* resulted in a small colony phenotype (Fig. 3C). We also tested the effect of knockdowns in TY broth using one sgRNA that caused a small colony phenotype (*atpD*) and one that did not (*atpF*). Interestingly, both knockdowns caused a strong growth defect in broth, but only if cultures were pre-grown overnight in 1% xylose to deplete the AtpD or AtpF proteins before sub-culturing (Fig. 3D). As an aside, we found that all four *atp* operon knockdowns were sensitized to subinhibitory concentrations of the uncoupler carbonyl cyanide m-chlorophenylhydrazone (CCCP, Fig. 3E), which hints at the potential for using our CRISPRi library to study drug targets in *C. difficile* (7, 12).

Three genes (*hisC*, *ilvB,* and *ilvC*) involved in amino acid biosynthesis were identified as essential despite the utilization of a growth medium rich in tryptone. These genes were not essential for Dembek et al. Note, however, that there are seven essential lysine biosynthesis genes, which we categorized under cell envelope rather than metabolism owing to their role in the synthesis of diaminopimelate for peptidoglycan. Finally, the global regulator CodY is essential by Tn-seq. CodY is widely conserved in Bacillota and senses GTP and branched-chain amino acids to regulate gene expression in response to the energetic and nutritional needs of the cell. In *C. difficile,* CodY represses hundreds of genes during exponential growth, and a *codY* null mutant grows poorly upon entry into stationary phase (128–130), which likely explains the Tn-seq result.

### Nucleotides and cofactors

We identified 11 genes essential for nucleotide biosynthesis. All 11 were also essential or ambiguous for Dembek et al., and 5 are essential in *B. subtilis* as well. One interesting difference is that an anaerobic ribonucleotide reductase encoded by *nrdD* and *nrdG* is essential in *C. difficile*, but these genes are not found in *B. subtilis*, which has instead an aerobic ribonucleotide reductase encoded by *nrdE* and *nrdF* that are not found in *C. difficile* (131). Two additional exceptions are *guaA* (GMP synthase) and *thyA* (thymidylate synthase), which are essential in *C. difficile* and *S. aureus* but not *B. subtilis* (14, 30, 33)*.* Two genes for regulatory nucleotides appear to be essential in *C. difficile*, the cyclic-di-AMP phosphodiesterase *yybT* and the bifunctional (pp)pGpp synthase/hydrolase *relA*. In *C. difficile, relA* is also called *rsh* and synthesizes exclusively pGpp (132, 133). The essentiality of *relA* was confirmed by CRISPRi (Table S1). The *B. subtilis* orthologs of *yybT* and *relA* are not essential (30), and their apparent essentiality in *C. difficile* could be due to polarity. However, in addition to the putatively essential bifunctional enzyme RelA, *C. difficile* contains a non-essential monofunctional pGpp synthetase RelQ (134). Based on analogy to other organisms, RelA could be essential in *C. difficile* because its hydrolase activity is needed to counterbalance the pGpp synthetase activity of RelQ (135, 136). As an aside, we note that cyclic-di-AMP is essential in *C. difficile* growing on rich media, but c-di-AMP synthases were not identified by Tn-seq because there are two of them, neither of which is individually essential (137).

In all, 24 genes are essential for the synthesis of cofactors, despite the utilization of media containing tryptone and yeast extract. All but two of these were also essential or ambiguous for Dembek et al., and 14 have an essential ortholog in *B. subtilis*. Curiously, neither we nor Dembek et al. scored dihydrofolate reductase (*dfrA*) as essential. Dihydrofolate reductase is the target of several important antibiotics and is essential in *E. coli*, *B. subtilis*, *S. sanguinis*, and *S. aureus* (30, 33, 47, 48).

### Phage and transposon-related genes

The *C. difficile* genome has a remarkably high content of mobile genetic elements (25, 138). Mobile genetic elements are not part of the core genome and thus should not be essential for viability. Nevertheless, 21 genes classified as essential appear to reside on a prophage or a transposon. Some of these might be false positives because only eight were also essential or ambiguous for Dembek et al. Even the eight genes classified as essential in both studies are likely due to indirect effects such as the induction of a lytic prophage.

### Transporters

Six genes for transporters were classified as essential in our Tn-seq, three of which were also essential for Dembek et al. and were confirmed by CRISPRi (Table S1). These encode a predicted Ktr potassium transporter and a predicted CorA-like divalent metal ion transporter. In *B. subtilis*, there are two Ktr systems, which are not essential but improve growth at high osmolarity (139).

### Sporulation

Curiously, both we and Dembek et al. classified the sporulation-associated phosphatases *ptpA* and *ptpB* as ambiguous or essential for vegetative growth. Two labs have reported null mutants of these genes, so they are not formally essential (34, 140, 141). Loss of *ptpA* or *ptpB* enhances sporulation, which we confirmed using CRISPRi against *ptpB* (Table S1). We presume that *ptp* genes are essential by Tn-seq because enhanced sporulation reduces vegetative growth.

### Genes of unknown function

Our Tn-seq analysis identified 28 putatively essential genes that could not be assigned to a functional pathway. None of these genes have an essential ortholog in *B. subtilis*, although in five cases BioCyc identified a non-essential ortholog. Eleven of these genes were not essential for Dembek et al. and in two cases (*cdr20291_3519* and *cdr20291_3520*) essentiality is likely due to polarity. That leaves 15 genes that are essential or ambiguous in two independent Tn-seq studies and are therefore likely to be *bona fide* essential genes. As noted above in the discussion of our CRISPRi experiments, we silenced expression of 11 of these genes and observed a viability defect for 9 of them, often accompanied by abnormal morphologies (Table 2, Table S1). The apparently essential genes of unknown function constitute a high-value gene set from the perspectives of bacterial physiology and antibiotic development.

### Conclusions

In summary, we identified 346 protein-encoding genes that, by Tn mutagenesis, are essential for vegetative growth of *C. difficile* strain R20291 on TY media. Of these, 283 were also identified as essential by Tn mutagenesis in a previous study (14), and 169 have an essential ortholog in *B. subtilis* (30). Overall, these results are broadly consistent with studies of gene essentiality in model organisms such as *E. coli*, *B. subtilis,* and *S. aureus* (30, 33, 46, 47, 59). The 283 *C. difficile* genes identified as essential in two independent Tn mutagenesis studies can be regarded as a consensus “essentialome” that minimizes false positives. Most of these genes play key roles in foundational cellular processes such as DNA replication, transcription, translation, and cell envelope biogenesis. But the consensus essentialome also includes 15 genes that could not be assigned to any functional pathway (Table 2, Table S3A and B). These genes might be targets for antibiotics that kill *C. difficile* without decimating the healthy microbiota needed to keep *C. difficile* in check.

We also used CRISPRi knockdown to investigate 181 genes that had been identified as essential in a previous transposon mutagenesis analysis (14). Our goals were to vet essentiality and screen for morphological defects that would facilitate assigning genes of unknown function to physiological pathways. Our CRISPRi platform used a plasmid that expresses *dCas9* from a xylose-inducible promoter (P_xyl_) and an sgRNA from a strong constitutive promoter (P_gdh_) (15). CRISPRi resulted in reduced plating efficiencies and/or small colony phenotypes on TY-xylose plates for 167 of the 181 genes targeted, a very high confirmation rate of 92%. The 14 genes for which no viability defect was observed could be false positives from the previous report or genes for which our sgRNAs were ineffective. Of these genes, 10 sustained insertions in our Tn-seq experiments, so we infer they are non-essential. Four did not sustain Tn insertions and are therefore likely to be essential genes that were poorly repressed by our sgRNAs. Importantly, no growth defects were observed using 20 control sgRNAs that did not target anywhere in the genome, indicating off-target effects are rare.

Microscopy of surviving cells scraped from the TY-xylose plates revealed that most knockdowns resulted in morphological abnormalities (151 out of 181 genes, 83%). Disappointingly, however, the utility of these defects for making functional assignments was limited by the observation that repressing genes of known function often resulted in non-intuitive defects. For example, repressing RNA polymerase gene *rpoB* resulted in severe filamentation suggestive of a cell division defect, while repressing the nucleotide biosynthesis gene *guaA* caused a chaining phenotype suggestive of a daughter cell separation defect. Non-intuitive phenotypes have also been reported in other CRISPRi screens (7, 8).

The findings and resources presented here should help guide future studies of *C. difficile*. First, our results can be used to prioritize genes for more rigorous but labor-intensive investigation using depletion strains with in-frame deletions (142). The 15 apparently essential genes that could not be assigned to a functional pathway seem like a good place to start. Second, our CRISPRi library can be leveraged to investigate antibiotic sensitivities (7, 12, 143), which might illuminate gene function and reveal vulnerabilities that can be exploited to improve treatment of *C. difficile* infections. Third, the identification of 18 proteins that localize to the midcell raises new questions related to *C. difficile* morphogenesis. For example, septal localization of the canonical elongation proteins MreC and MreD suggests they contribute to cell division, and/or *C. difficile* elongates by inserting new peptidoglycan near the midcell. In addition, our discovery that YlmG, YlxW, and YlxX localize to the division site provides the most direct evidence to date that these conserved but enigmatic proteins play a role in cell division.

## MATERIALS AND METHODS

### Strains, media, and growth conditions

Most bacterial strains used in this study are listed in Table S4. Strains and plasmids constructed for the CRISPRi library are summarized separately in Table S2. *C. difficile* strains were derived from R20291 (144). *C. difficile* was routinely grown in tryptone-yeast extract (TY) medium, supplemented as needed with thiamphenicol at 10 µg/mL (TY-Thi10). TY medium consisted of 3% tryptone, 2% yeast extract, and 2% agar (for plates). Brain heart infusion (BHI) media was prepared per manufacturer’s (DIFCO) instructions. *C. difficile* strains were maintained at 37°C in an anaerobic chamber (Coy Laboratory Products) in an atmosphere of 2% H_2_, 5% CO_2_, and 93% N_2_. *Escherichia coli* strains were grown in LB medium at 37°C with chloramphenicol at 10 µg/mL and/or ampicillin at 100 µg/mL as needed. LB medium contained 1% tryptone, 0.5% yeast extract, 0.5% NaCl, and 1.5% agar (for plates). OD_600_ measurements were made with the WPA Biowave CO8000 tube reader in the anaerobic chamber.

### Plasmid and strain construction

Plasmids are listed in Table S5 and were constructed with HiFi DNA Assembly from New England Biolabs (Ipswich, MA). Oligonucleotide primers (Table S6) were synthesized by Integrated DNA Technologies (Coralville, IA). CRISPRi plasmids were constructed as described in reference (15). Regions constructed by PCR were verified by DNA sequencing. Plasmids were propagated in *E. coli* HB101/pRK24 and conjugated into *C. difficile* R20291 according to reference (54). Final R20291 CRISPRi strains were verified by PCR amplifying and sequencing the guide region. Details relevant to other plasmid construction are provided in Table S5.

### CRISPRi screen

Overnight cultures grown in TY-Thi10 were serially diluted 10-fold in TY, and 5 µL were spotted on TY-Thi10 and TY-Thi10 1% (wt/vol) xylose plates. Plates were incubated at 37°C overnight and imaged the following morning (~18 h). Cells were scraped from select spots (usually the last spot with growth) and resuspended in 50 µL TY. Cell suspensions were supplemented with 5 µg/mL FM4-64 (red fluorescent membrane stain, Thermo Scientific) and 15 µg/mL Hoechst 33342 (blue fluorescent DNA stain, Invitrogen) and imaged by phase-contrast and fluorescence microscopy.

### Protein localization

R20291 harboring plasmids that expressed RFP-tagged proteins under xylose control were grown in TY-Thi10 overnight, subcultured into TY-Thi10 with 0.1% or 1% xylose, grown to an OD_600_ of about 0.6, and fixed with 4% buffered paraformaldehyde as described (54, 122, 145). Fixed cells were photographed under phase-contrast and (red) fluorescence. Septal localization was scored manually by inspecting cells for the presence of a fluorescent band near the midcell. MicrobeJ was used to keep track of cells that scored positive or negative for septal localization (146).

### Microscopy

Cells were immobilized using thin agarose pads (1% wt/vol agarose). Phase-contrast micrographs were recorded on an Olympus BX60 microscope equipped with a 100× UPlanApo objective (numerical aperture, 1.35). Micrographs were captured with a Hamamatsu Orca Flash 4.0 V2+ complementary metal oxide semiconductor (CMOS) camera. Excitation light was generated with an X-Cite XYLIS LED light source. Red fluorescence was detected with the Chroma filter set 49008 (538–582 nm excitation filter, 587 nm dichroic mirror, and a 590–667 nm emission filter). Blue fluorescence was detected with the Olympus filter set U-MWU (330–385 nm excitation filter, 400 nm dichroic mirror, and a 420 nm barrier emission filter).

### Transposon library construction

Plasmid pRPF215 is a quasi-suicide plasmid that harbors the *Himar1 mariner* transposase gene under control of P*_tet_* (14). The gene for TetR does not have a terminator, and transcription reads through into the origin of replication, presumably disrupting plasmid replication. The addition of anhydrotetracycline therefore both induces the transposase and causes plasmid loss. A single colony of R20291/pRPF215 was used to inoculate a 2 mL overnight culture in TY-Thi10. In total, 20 independent overnight cultures were grown for each transposon library construction. After overnight growth, each was then sub-cultured 1:50 into 2 mL TY and grown to an OD_600_ of 0.3. From each subculture, an aliquot was removed and spread onto two large (15 cm diameter) plates of TY agar with 80 µg/mL lincomycin (RPI) and 100 ng/mL anhydrotetracycline (Sigma), for a total of 40 plates. We used higher concentrations of lincomycin than originally published (14) because we found 80 µg/mL lincomycin decreased the number of false positives. The amount of subculture to plate was experimentally determined to give roughly 5,000–8,000 colonies. Typically, we used 220 µL of subculture diluted with TY to 600 µL, a volume suitable for spreading evenly on a large plate. A dilution series of one subculture was also plated on TY to calculate plating efficiency. Selection plates typically grew one colony for every 500 plated (i.e., an efficiency of about 2 × 10^−3^). Plates were incubated for 20 hours at 37°C. Cells were then scraped off the plates with 5 mL TY each, pooled, amended to 10% DMSO, aliquoted, and stored at 80°C. This material was referred to as the primary transposon library. Suspensions of the primary libraries typically had an OD_600_ of about 6. The concentration of viable cells was quantified by plating aliquots on TY plates and was typically around 3 × 10^8^ CFU/mL. Three independent libraries were constructed on different days.

### Tn-seq sample preparation

DNA samples were prepared directly from 1 mL of primary library or from 10 mL culture that had been grown for an additional seven doublings in TY. To avoid creating a bottleneck, 10 mL TY was inoculated with 2.2 × 10^7^ CFU. There are 502,945 possible TA insertion sites in the R20291 chromosome; thus, cultures were started with a ratio of about 45 CFU per TA site. DNA libraries for Illumina sequencing were prepared based on modifications of Karash et al. (26). Briefly, regions adjacent to any transposon insertion were amplified by single primer extension. The resulting products were extended with a cytosine tail, which then allowed further amplification by PCR. The upstream primer recognizes the transposon sequence, incorporates the P5 sequence for Illumina sequencing and a sample-specific barcode; the downstream primer recognizes the C-tail and incorporates the P7 sequence.

Genomic DNA was prepared using the Monarch Genomic DNA purification kit from NEB, using the protocol for Gram-positive bacteria. A maximum of 2 × 10^9^ cells were pelleted. Lysis was facilitated through the addition of 0.5 mg hen egg white lysozyme (Boehringer Mannheim) and 20 U mutanolysin (Sigma), and DNA was eluted in 35 µL with a typical yield of 200 ng/µL. Linear extension PCR was performed on 100 ng DNA in 50 µL with Taq polymerase (NEB) and primer Tn-ermB-2 (anneal: 30 s at 55°C, extend 30 s at 68°C, 50 cycles). The resulting product was spin-column purified (Zymo Research Clean & Concentrator kit) and eluted in 12 µL. A C-tail was added by extending with terminal transferase (NEB) in a 20 µL reaction, using 1.25 mM dCTP (NEB) and 50 µM ddCTP (MilliporeSigma/Roche). The product was again spin-column purified and eluted in 10 µL. Final PCR amplification used 1 µL of C-tailed DNA in a 35 µL reaction mixture, Taq polymerase, and primers P716G and P5TnPx (x: variable barcode; anneal: 30 s at 62°C, extend 30 s at 68°C, 35 cycles). The resulting product was separated on a 1.5% agarose gel in Tris Acetate EDTA buffer (TAE). Fragments of 300–500 base pair length were excised, purified with the Zymo Research Gel DNA recovery kit, and eluted in 10 µL. DNA concentration was quantified with the Qubit dsDNA assay and was typically around 5 ng/µL. Four samples with distinct barcodes were combined and submitted for sequencing (Illumina HiSeq X, 150 bp PE reads) with Admera Health Biopharma Services (South Plainfield, NJ). Samples were spiked with 5% PhiX DNA to improve data quality.

### Sequencing data processing

Raw sequencing files were first trimmed with Trimmomatic to eliminate poor-quality reads (147). The first four bases before the barcodes were then removed using Trim Sequences, and the resulting files were de-multiplexed using the Barcode splitter, both on Galaxy (148). Reads were aligned to the reference genome of R20291 (NC_013316.1 or ASM2710v1) using the Burrows-Wheeler Aligner (BWA) provided in TRANSIT (28). Finally, the resulting Wig files were compared in TRANSIT2, which evaluates gene essentiality both by Gumbel analysis and binomial analysis (149). The former makes essentiality calls based on insertion gaps, that is, consecutive TA sites lacking transposon insertions, using the Gumbel distribution (150). The latter calls essentiality for small genes lacking insertions, which can be difficult to detect by the more conservative Gumbel algorithm (29). Essentiality calls are either “E” when identified by Gumbel or “EB” when identified by the Binomial analysis. Table S3 lists genes that were called essential in primary insertion libraries using cells scraped from plates, or after an additional 7 generations of growth. The library data set was generated from three independently constructed transposon libraries. The outgrowth data set was generated from two independent growth cultures from each of the three independent libraries. We present both the separate data output as well as a combined essentiality call (Table S3). The latter was further hand-edited by including 11 genes (indicated as “Ei” for “essential by inspection”) that appeared to have been mistakenly called non-essential by TRANSIT2. Ten of these genes had very few insertions despite numerous possible TA sites, while the eleventh had a large number of insertions but mostly at the 3′ end of the gene.

## Data Availability

CRISPRi strains from this study are available as *E. coli* conjugation donors by request from the corresponding authors. All raw Tn-seq sequencing data have been deposited with NCBI Sequence Read Archive (SRA) under BioProject ID PRJNA1295396 (BioSamples SAMN50168355-SAMN50168363).

## SUPPLEMENTAL MATERIAL

Supplemental figures, tables, and references jb.00220-25-s0001.pdf Fig. S1- and S2, Tables S4 to S6, and supplemental references.

Table S1 jb.00220-25-s0002.xlsx CRISPRi screen.

Table S2 jb.00220-25-s0003.xlsx CRISPRi strains and plasmids.

Table S3 jb.00220-25-s0004.xlsx Genes identified as essential by Tn-seq.

ASM does not own the copyrights to Supplemental Material that may be linked to, or accessed through, an article. The authors have granted ASM a non-exclusive, world-wide license to publish the Supplemental Material files. Please contact the corresponding author directly for reuse.
