# Supplementary material for: Analysis of essential genes in Clostridioides difficile by CRISPRi and Tn-seq
Source: J Bacteriol. 2025 Sep 8;207(10):e00220-25. doi: 10.1128/jb.00220-25 (PMC12548448; doi:10.1128/jb.00220-25)
Supplement: Supplemental figures, tables, and references — Fig. S1- and S2, Tables S4 to S6, and supplemental references. [file jb.00220-25-s0001.pdf]

Supplemental information for:

Analysis of Essential Genes in *Clostridioides difficile* by CRISPRi and Tn-seq

Maia E. Alberts[1,3], Micaila P. Kurtz[1], Ute Müh[1], Jonathon P. Bernardi[1], Kevin W. Bollinger[1], Horia A. Dobrila[1,4], Leonard Duncan[1], Hannah M. Laster[1], Andres J. Orea[1,5], Anthony G. Pannullo[1,6], Juan G. Rivera-Rosado[1], Facundo V. Torres[1,7], Craig D. Ellermeier[1,2]*, David S. Weiss[1,2]*

[1]Department of Microbiology and Immunology, Carver College of Medicine, University of Iowa, Iowa City, IA, USA

[2]Graduate Program in Genetics, University of Iowa, Iowa City, IA, USA

[3]Present address: Department of Anesthesiology, Carver College of Medicine, University of Iowa, Iowa City, IA, USA.

[4]Present address: Departments of Medicine and Medical Microbiology & Immunology, University of Wisconsin, Madison, WI, USA

[5]Present address: Department of Pharmacology, University of California, Davis, CA, USA

[6]Present address: JMI Laboratories, North Liberty, IA, USA

[7]Present address: Weill Institute for Cell & Molecular Biology, Cornell University, Ithaca, NY, USA

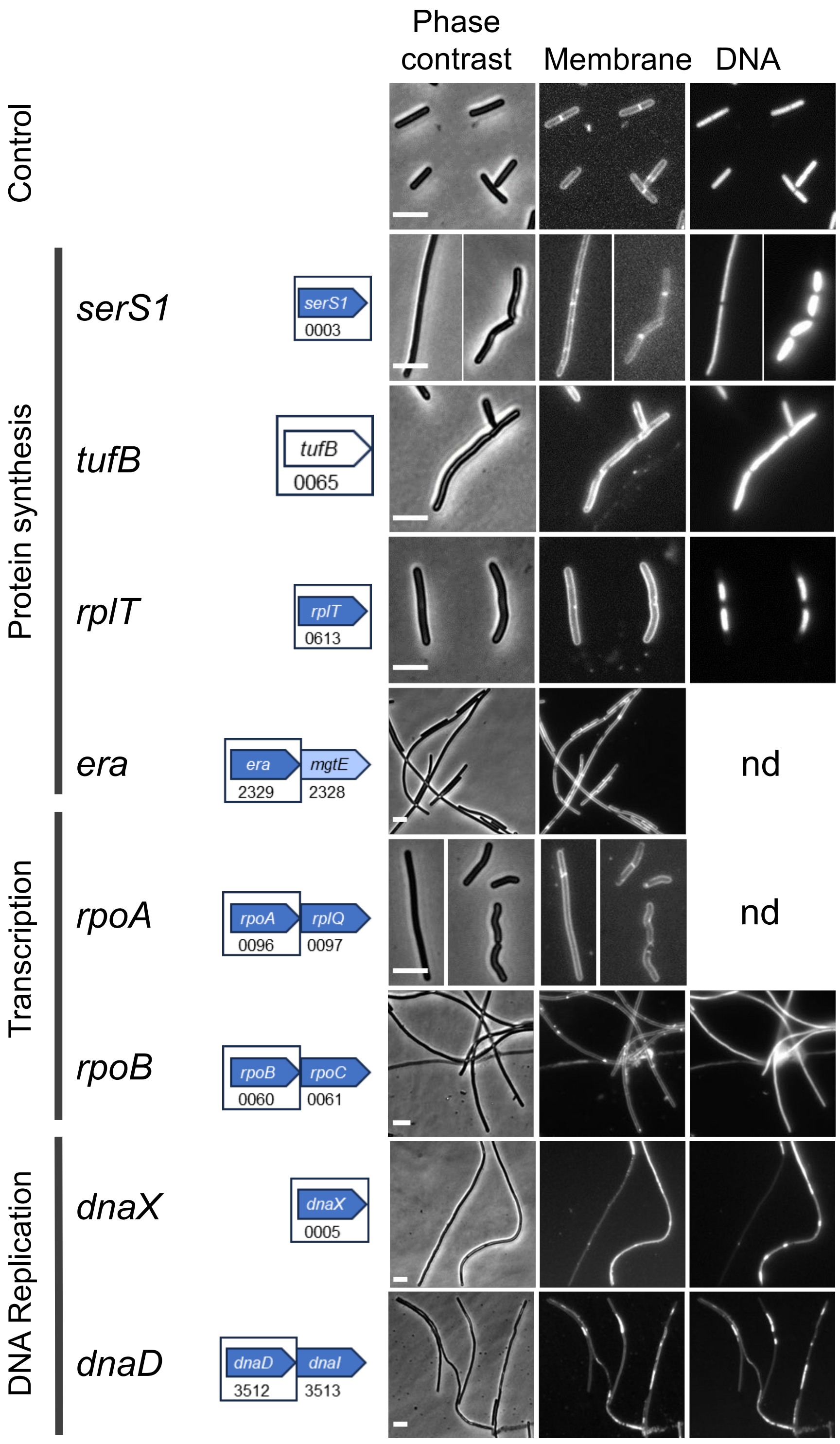
Phase contrast
Membrane
DNA
Control
Protein synthesis
serS1
serS1
0003
tufB
tufB
0065
rplT
rplT
0613
era
era
2329
mgtE
2328
nd
Transcription
rpoA
rpoA
0096
rplQ
0097
nd
rpoB
rpoB
0060
rpoC
0061
DNA Replication
dnaX
dnaX
0005
dnaD
dnaD
3512
dnaI
3513

Fig S1

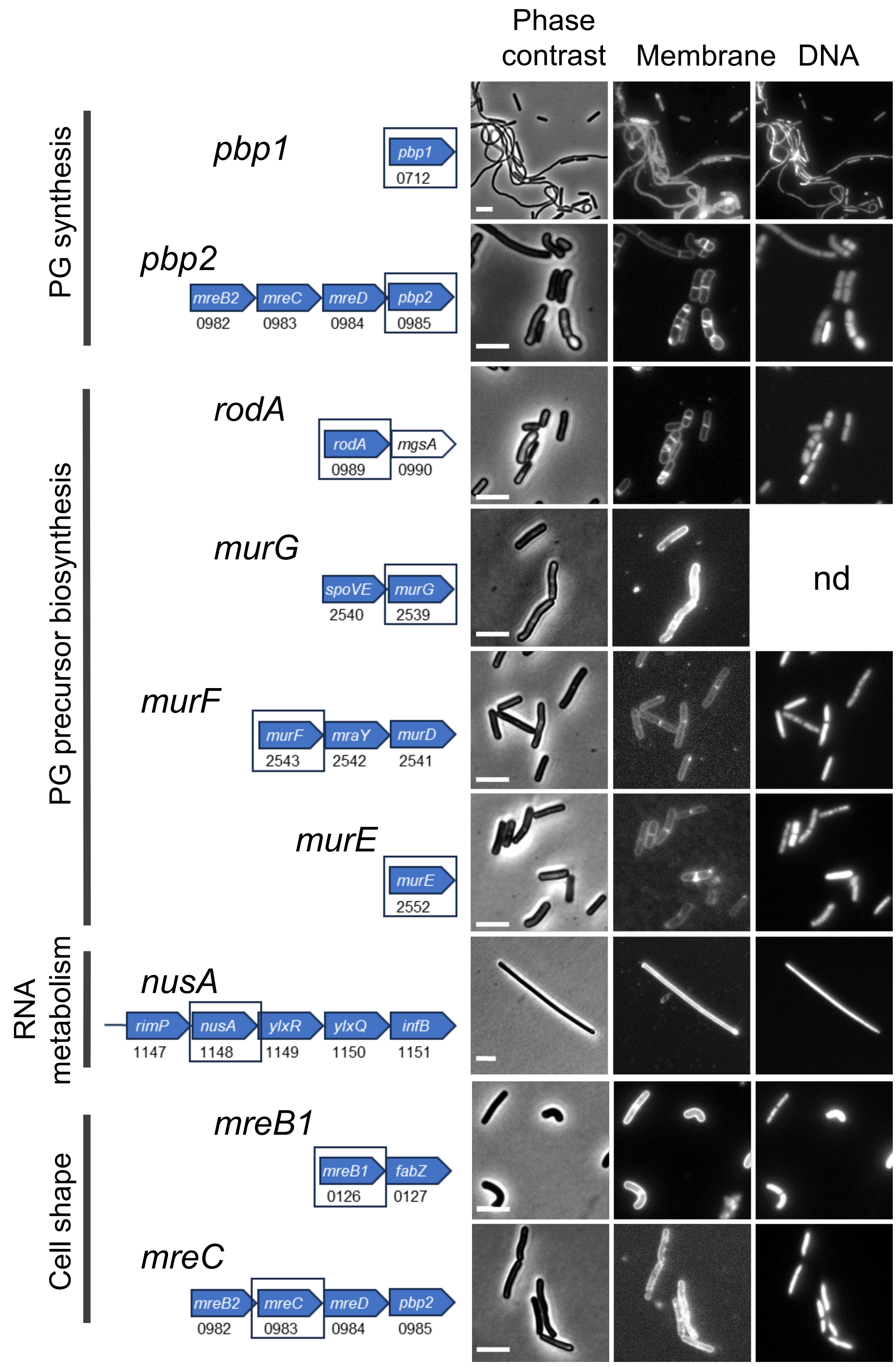

Phase contrast
Membrane
DNA
PG synthesis
pbp1
pbp1
0712
pbp2
mreB2
mreC
mreD
pbp2
0982
0983
0984
0985
PG precursor biosynthesis
rodA
rodA
mgsA
0989
0990
murG
spoVE
murG
2540
2539
nd
murF
murF
mraY
murD
2543
2542
2541
murE
murE
2552
RNA metabolism
nusA
rimP
nusA
ylxR
ylxQ
infB
1147
1148
1149
1150
1151
Cell shape
mreB1
mreB1
fabZ
0126
0127
mreC
mreB2
mreC
mreD
pbp2
0982
0983
0984
0985

Fig S1, cont.

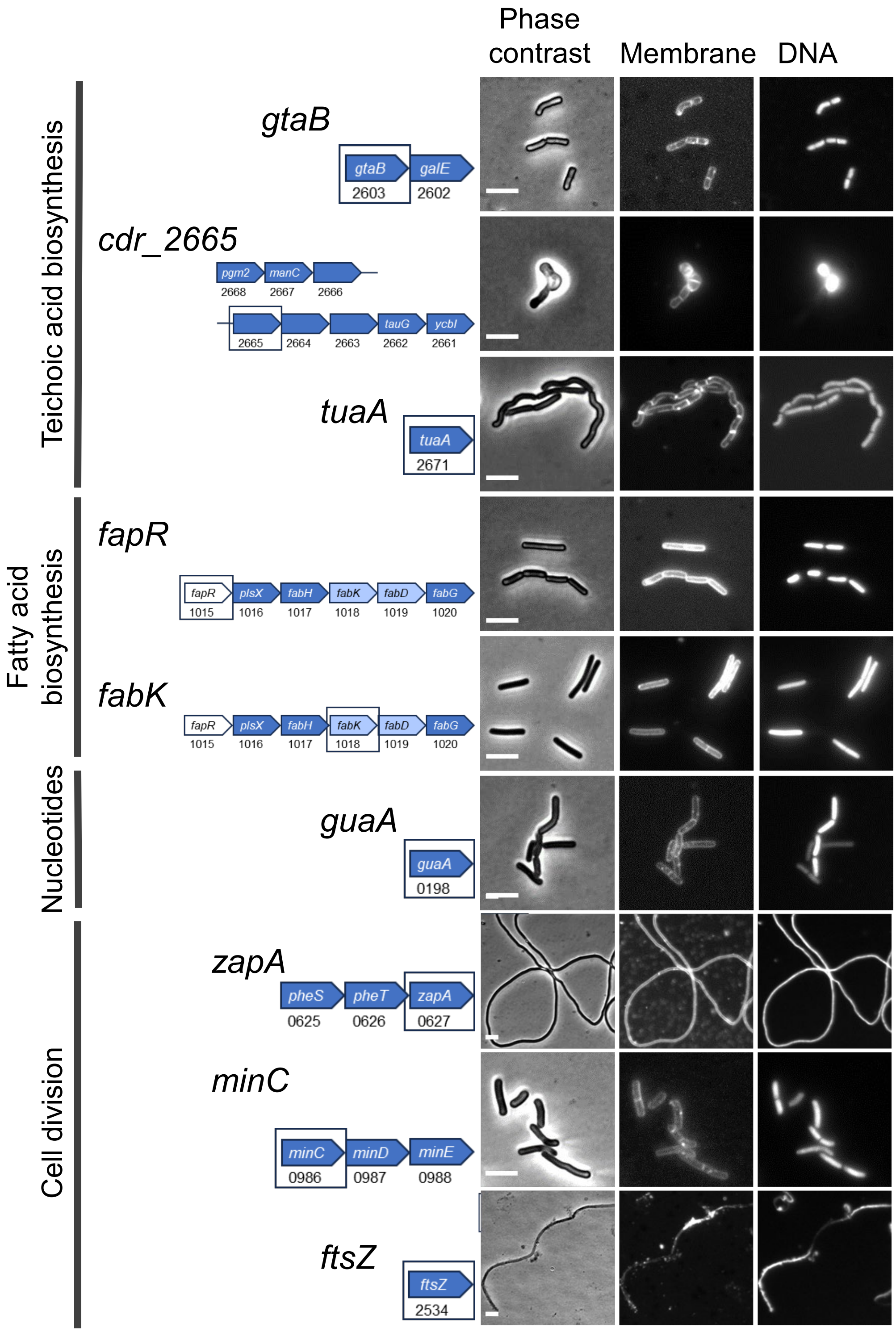
Phase contrast
Membrane
DNA
Teichoic acid biosynthesis
gtaB
gtaB 2603
galE 2602
cdr_2665
pgm2 2668
manC 2667
2666
2665
2664
2663
tauG 2662
ycbI 2661
tuaA
tuaA 2671
Fatty acid biosynthesis
fapR
fapR 1015
plsX 1016
fabH 1017
fabK 1018
fabD 1019
fabG 1020
fabK
Nucleotides
guaA
guaA 0198
Cell division
zapA
pheS 0625
pheT 0626
zapA 0627
minC
minC 0986
minD 0987
minE 0988
ftsZ
ftsZ 2534

Fig S1, cont.

Fig. S1. Extended version of Figure 2, morphology of CRISPRi strains with sgRNAs targeting genes in select functional pathways. Left: pathway. Middle: Predicted transcription unit. Targeted genes are boxed and indicated above the operon diagrams. Numbers are R20291 locus tags. Genes are color-coded to indicate essentiality based on Tn-seq calls in Table S3. Dark blue: essential. Light blue: ambiguous. White: non-essential. Operon structure is not to scale. Right: Morphological changes based on phase contrast and fluorescence micrographs of cells scraped from viability plates. Membranes were stained with FM4-64 and DNA was stained with Hoechst 33342. n.d. means not determined. Size bars are 5 µm. Micrographs are representative of at least two experiments. The control strain expressed an sgRNA that does not target anywhere in the genome. Note: *tufB* is essential by CRISPRi but not by Tn-seq because the sgRNA against *tufB* also represses *tufA*.

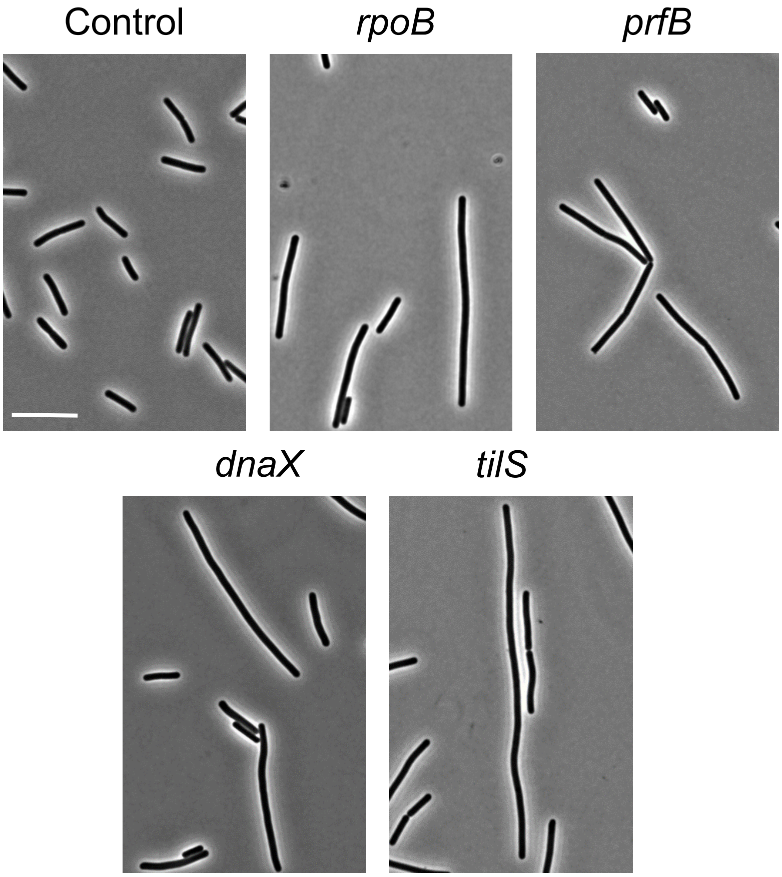

Fig. S2. Morphological phenotypes induced by CRISPRi against select genes in TY broth. Starter cultures of the indicated CRISPRi strains that had been grown overnight in TY-Thi10 were diluted 1:400 into fresh TY-Thi10 with 1% xylose and grown for 5.5h (~ 6 mass doublings) to OD_{600} ~0.35. Cells were fixed and photographed under phase contrast. Size bar = 5 µm. The control strain expressed an sgRNA against a sequence not present in R20291. Images are representative of at least two experiments.

Table S4: Strains

**Strain**	**Genotype**	**Comment**	**Source**
E. coli			
HB101/pRK24	F− *mcrB mrr hsdS20* (r_B^- m_B^-) *recA13 leuB6 ara-14 proA2 lacY1 galK2 xyl-5 mtl-1 rpsL20*		Dineen et al. 2007, Trieu-Cuot et al. 1991
C. difficile			
R20291	Wild-type *C. difficile* strain from UK outbreak (ribotype 027)		
UM1320	R20291/pCE1125	*Pxyl::rfp-pbp1 catP*	This study
UM1175	R20291/pCE1126	*Pxyl::rfp-pbp2 catP*	This study
KB412	R20291/pHL07	*Pxyl::rfp-ylxX catP*	This study
KB413	R20291/pHL08	*Pxyl::rfp-ylxW catP*	This study
KB418	R20291/pHL09	*Pxyl::rfp-ftsQ catP*	This study
KB448	R20291/pHL13	*Pxyl::rfp-cdr_3331 catP*	This study
KB449	R20291/pHL14	*Pxyl::rfp-ldt4 catP*	This study
KB455	R20291/pHL15	*Pxyl::rfp-ldt5 catP*	This study
KB454	R20291/pHL24	*Pxyl::rfp-ftsK catP*	This study
UM0359	R20291/pIA34	*pxylR::dCas9-opt Pgdh::sgRNA-neg catP*	Müh et al. 2019
UM1247	R20291/pJR01	*Pxyl::divIVA-rfp catP*	This study
UM1251	R20291/pJR05	*Pxyl::rfp-ylmG catP*	This study
UM1252	R20291/pJR06	*Pxyl::sepF-rfp catP*	This study
UM1254	R20291/pJR17	*Pxyl::rfp-mreC catP*	This study
UM1255	R20291/pJR18	*Pxyl::rfp-mreD catP*	This study
UM1258	R20291/pJR23	*Pxyl::rfp-mgt catP*	This study
LD0110	R20291/pLD01	*Pxyl::rfp-ldt1 catP*	This study
LD0122	R20291/pLD07	*Pxyl::rfp-ftsZ catP*	This study
UM1420	R20291/pMK50	*Pxyl::rfp-mrdB catP*	This study
CDE2716	R20291/pRPF215	*Ptet-himar1-Ter(slpA)-transposon*	Dembek et al. 2015

Table S5: Plasmids

Plasmid	Relevant features	Parent vector	Restriction enzymes to digest parent vector	PCR primers	PCR template	Assembly	Comments	Reference
pAP114	*Pxyl::rfp*						Genbank: MK368760	Müh et al. 2019
pCE1125	*Pxyl::rfp-pbp1*	pDSW2040	KpnI, SacI	6660+6661	pAP114	HiFi[a]		
pCE1126	*Pxyl::rfp-pbp2*	pDSW2041	KpnI, SacI	6660+6661	pAP114	HiFi		
pDSW1728	*Ptet::rfp*						Genbank: KT371995	Ransom et al. 2015
pDSW2021	*Pxyl::* MCS-*rfp*	pAP114	SacI, BamHI	P2382+P2383	pDSW1728	Ligation		
pDSW2040	*Ptet:rfp-pbp1*	pRAN473	SphI, BamHI	P2401+P2402	R20291	Ligation		
pDSW2041	*Ptet:rfp-pbp2*	pRAN473	SphI, BglII	P2303+P2304	R20291	Ligation		
pHL07	*Pxyl::rfp-cdr_2536(ylxX)*	pMK02	KpnI, BamHI	6687+6688	R20291	HiFi		
pHL08	*Pxyl::rfp-cdr_2537(ylxW)*	pMK02	KpnI, BamHI	6689+6690	R20291	HiFi		
pHL09	*Pxyl::rfp-cdr_2538(ftsQ)*	pMK02	KpnI, BamHI	6691+6692	R20291	HiFi		
pHL13	*Pxyl::rfp-cdr_3331*	pMK02	KpnI, BamHI	6699+6700	R20291	HiFi		
pHL14	*Pxyl::rfp-cdr_1285(ldt4)*	pMK02	KpnI, BamHI	6701+6702	R20291	HiFi		
pHL15	*Pxyl::rfp-cdr_2055(ldt5)*	pMK02	KpnI, BamHI	6703+6704	R20291	HiFi		
pHL24	*Pxyl::rfp-cdr_1165(ftsK)*	pMK02	KpnI, BamHI	6721+6722	R20291	HiFi		
pIA131	*Pxyl::* MCS-*rfp*	pDSW2021	KpnI, BamHI	6786+6788	pMK02	HiFi	Corrects mutation in pDSW2021	
pIA33	*p_{xyl} R::dCas9-opt P_{gdh} ::sgRNA-rfp catP*						Parent plasmid for CRISPRi constructs; Genbank MK368761	Müh et al. 2019
pIA34	*p_{xyl} R::dCas9-opt P_{gdh} ::sgRNA-neg catP*						Negative control CRISPRi plasmid	Müh et al. 2019
pJR01	*Pxyl::cdr_2503(divIVA)-rfp*	pIA131	KpnI, SacI	6675+6676	R20291	HiFi		
pJR05	*Pxyl::rfp-cdr_2505(ylmG)*	pMK02	KpnI, BamHI	6683+6684	R20291	HiFi		
pJR06	*Pxyl::cdr_2506(sepF)-rfp*	pIA131	KpnI, SacI	6685+6686	R20291	HiFi		
pJR17	*Pxyl::rfp-cdr_0983(mreC)*	pMK02	KpnI, BamHI	6707+6708	R20291	HiFi		
pJR18	*Pxyl::rfp-cdr_0984(mreD)*	pMK02	KpnI, BamHI	6709+6710	R20291	HiFi		
pJR23	*Pxyl::rfp-cdr_2283(mgt)*	pMK02	KpnI, BamHI	6731+6732	R20291	HiFi		
pLD1	*Pxyl::rfp-cdr_2797(ldt1)*	pMK02	KpnI, BamHI	6938+6939	R20291	HiFi		
pLD7	*Pxyl::rfp-cdr_2534(ftsZ)*	pMK02	KpnI, BamHI	6998+6999	R20291	HiFi		
pMK02	*Pxyl::rfp-cdr_0018*	pCE1125	SphI-BamHI	6578+6579	R20291	HiFi		
pMK50	*Pxyl::rfp-cdr_0989(rodA)*	pMK02	KpnI, BamHI	7672+7673	R20291	HiFi		
pRAN473	*Ptet::rfp-* MCS							Ransom et al. 2015
pRPF215	Ptet-himar1-Ter(slpA)-transposon							Dembek et al. 2015

[a]: HiFi assembly (NEB)

Table S6: Oligonucleotides

Oligo	Sequence	Comment
CDEP6578	TATGGATGAATTATATAAAATGAACGGTGGTGGTGGTGGTACCTTGATAAGGAAAGTAACAAGAATATTG	
CDEP6579	ATTTAAAGTTTTATTAAAACTTATAGGATCCTTAATTTTTAGGTTTTGCAAATATCATTC	
CDEP6660	GCTTCTTATTTTTATGGTACCGTATAATTTAAAGGGTTATGGCTGT	
CDEP6661	TCTCCTTTACTGCAGGAGCTCACTTCATAACTATCGTTTTCCT	
CDEP6675	CGATAGTTATGAAGTGAGCTCAAGGAGAAAATTTTATGCTAACTCCAATTGAGATAGAAAATAAAG	
CDEP6676	AGATACCATAGATCCGGTACCAGATCCAGATCCTTCTAAAGTTGTAGCAGCTTCATCATTACTATA	
CDEP6683	AACGGTGGTGGTGGTGGTACCATGGGAACAATAAGAATTGCAC	
CDEP6684	ATTTAAAGTTTTATTAAAACTTATAGGATCCTTAGAATATAACCAATACTAATTGTCTTAGG	
CDEP6685	CGATAGTTATGAAGTGAGCTCAAGGAGAAAATTTTATGTCAAATGGAATAATATCTAAATTTAAGAATTG	
CDEP6686	AGATACCATAGATCCGGTACCAGATCCAGATCCTTTATTTTGCCAAGGGAAGAATG	
CDEP6687	AACGGTGGTGGTGGTGGTACCATGAAAAAGAAAATAGTTATATTAGGAGCA	
CDEP6688	ATTTAAAGTTTTATTAAAACTTATAGGATCCTTATTTTGCAAATAAATTCACTTTATTCCC	
CDEP6689	AACGGTGGTGGTGGTGGTACCTTGAAAAAAAGATTTATAAATAAAAGTATAATCATAGG	
CDEP6690	ATTTAAAGTTTTATTAAAACTTATAGGATCCTTATTTCCCCTCTGCTTTGTTTATATC	
CDEP6691	AACGGTGGTGGTGGTGGTACCTTGAAAAAAAGAAGAAAGTTAAATACGAATAATG	
CDEP6692	ATTTAAAGTTTTATTAAAACTTATAGGATCCCTATCCTTCTGGACTATACACTGC	
CDEP6699	AACGGTGGTGGTGGTGGTACCATGGAACAGAACATAACTTTGAAAG	
CDEP6700	ATTTAAAGTTTTATTAAAACTTATAGGATCCCTATCTAAATACCTTTTTCAAGAAACTC	
CDEP6701	AACGGTGGTGGTGGTGGTACCATGAGTAATGTGAACAAAAAGCTAG	
CDEP6702	ATTTAAAGTTTTATTAAAACTTATAGGATCCTTAAGCTTGCGGTTGTGG	
CDEP6703	AACGGTGGTGGTGGTGGTACCATGGGCAGAAGAACAACAAG	
CDEP6704	ATTTAAAGTTTTATTAAAACTTATAGGATCCCTATTTTTTTAATTCTTTATAAGAACTATTTGATATATG	
CDEP6707	AACGGTGGTGGTGGTGGTACCTTGGTGATGGCCTTGAGATTTG	
CDEP6708	ATTTAAAGTTTTATTAAAACTTATAGGATCCTTATCCTATATTTCTTGGTTCTATGACTA	
CDEP6709	AACGGTGGTGGTGGTGGTACCATGAAAAAAGTTTTACTTTGTCTGTTG	
CDEP6710	ATTTAAAGTTTTATTAAAACTTATAGGATCCCTAATCTTCTTTAAGTTTTAAAACTGCTC	
CDEP6721	AACGGTGGTGGTGGTGGTACCATGGCCACGAAAAAGAAGAA	
CDEP6722	ATTTAAAGTTTTATTAAAACTTATAGGATCCCTATTCACCTTCTAAATTCTGCAAATC	
CDEP6731	AACGGTGGTGGTGGTGGTACCATGGGTTATTACAATGATAATGATAATAATAAAATAAG	
CDEP6732	ATTTAAAGTTTTATTAAAACTTATAGGATCCTTAATCAATTTCTGATTTAAATGGAGTG	
CDEP6786	CTCTCTAGAGTCGACGGTACCGGATCTATGGTATCTAAAGGAGAAGAAGATAATATG	
CDEP6788	CTTTTTCTATTTAAAGTTTTATTAAAACTTATAGGATCCTTATTTATATAATTCATCCATACCTCCTGTTG	
CDEP6938	AACGGTGGTGGTGGTGGTACCATGATTGATGGAAAAGAGGTAAAAC	
CDEP6939	ATTTAAAGTTTTATTAAAACTTATAGGATCCTAGTATAAAATAATTGGTGTACCTGG	
CDEP6998	AACGGTGGTGGTGGTGGTACCATGATGCTAAACTTTGACGTAGA	
CDEP6999	ATTTAAAGTTTTATTAAAACTTATAGGATCCTTATCTTCTTCTTCTTAGGAATGTAGG	
CDEP7672	AACGGTGGTGGTGGTGGTACCTTGAAAAAAATCAATATTAATTATAAGTCGAC	
CDEP7673	TTTAAAGTTTTATTAAAACTTATAGGATCTTAGAAATTTATTTTCTTTCTTCTCATACAC	
P2303	CACGCATGCGGTGGATCTGAAGAAAATAAACAAGTTGATAGACTAAAAATAATTAAG	
P2304	CACAGATCTAAGCTATCTATTAATTTGTGACTCG	
P2401	CAC GCATGCGGTGGATCTAATAACGATAATGATAAAAATGGAAATAAAATCAGG	
P2402	CACGGATCCTTAAGTTGCTGGAGTTCCACC	
P5 Tn Px -	AATGATACGGCGACCACCGAGATCTACACTCTTTCCCTACACGACGCTCTTCCGATCTNNNNAGATCAC GCTGTCAGACCGGGGACTTATCAGCCAACCT	**NNNN**: variable barcode
P7 16G	CAAGCAGAAGACGGCATACGAGCTCTTCCGATCTGGGGGGGGGGGGGGGG	
Tn-ermB-2	ATCACTCCTTCTTAATTACAAATTTTTAGCATCTAATTTAACTTCAATTCCTATTATAC	

References Supplemental Information

1. Dembek M, Barquist L, Boinett CJ, Cain AK, Mayho M, Lawley TD, Fairweather NF, Fagan RP. 2015. High-throughput analysis of gene essentiality and sporulation in *Clostridium difficile*. MBio 6:e02383.
2. Dineen SS, Villapakkam AC, Nordman JT, Sonenshein AL. 2007. Repression of *Clostridium difficile* toxin gene expression by CodY. Mol Microbiol 66:206-19.
3. Edwards AN, Wetzel D, DiCandia MA, McBride SM. 2022. Three Orphan Histidine Kinases Inhibit *Clostridioides difficile* Sporulation. J Bacteriol 204:e0010622.
4. Elfmann C, Dumann V, van den Berg T, Stülke J. 2025. A new framework for SubtiWiki, the database for the model organism *Bacillus subtilis*. Nucleic Acids Res 53:D864-d870.
5. Koo BM, Kritikos G, Farelli JD, Todor H, Tong K, Kimsey H, Wapinski I, Galardini M, Cabal A, Peters JM, Hachmann AB, Rudner DZ, Allen KN, Typas A, Gross CA. 2017. Construction and Analysis of Two Genome-Scale Deletion Libraries for *Bacillus subtilis*. Cell Syst 4:291-305.e7.
6. Müh U, Pannullo AG, Weiss DS, Ellermeier CD. 2019. A Xylose-Inducible Expression System and a CRISPR Interference Plasmid for Targeted Knockdown of Gene Expression in *Clostridioides difficile*. J Bacteriol 201:e00711-18.
7. Ransom EM, Ellermeier CD, Weiss DS. 2015. Use of mCherry Red fluorescent protein for studies of protein localization and gene expression in *Clostridium difficile*. Appl Environ Microbiol 81:1652-60.
8. Shrestha S, Taib N, Gribaldo S, Shen A. 2023. Diversification of division mechanisms in endospore-forming bacteria revealed by analyses of peptidoglycan synthesis in *Clostridioides difficile*. Nat Commun 14:7975.
9. Trieu-Cuot P, Carlier C, Poyart-Salmeron C, Courvalin P. 1991. Shuttle vectors containing a multiple cloning site and a lacZ alpha gene for conjugal transfer of DNA from *Escherichia coli* to gram-positive bacteria. Gene 102:99-104.
10. Underwood S, Guan S, Vijayasubhash V, Baines SD, Graham L, Lewis RJ, Wilcox MH, Stephenson K. 2009. Characterization of the sporulation initiation pathway of *Clostridium difficile* and its role in toxin production. J Bacteriol 191:7296-305.
11. van Eijk E, Paschalis V, Green M, Friggen AH, Larson MA, Spriggs K, Briggs GS, Soultanas P, Smits WK. 2016. Primase is required for helicase activity and helicase alters the specificity of primase in the enteropathogen *Clostridium difficile*. Open Biol 6.
